# Submesoscale currents in the ocean

**DOI:** 10.1098/rspa.2016.0117

**Published:** 2016-05

**Authors:** James C. McWilliams

**Affiliations:** Department of Atmospheric and Oceanic Sciences, University of California, Los Angeles, CA 90095-1565, USA

**Keywords:** submesoscale, turbulence, frontogenesis, instability

## Abstract

This article is a perspective on the recently discovered realm of submesoscale currents in the ocean. They are intermediate-scale flow structures in the form of density fronts and filaments, topographic wakes and persistent coherent vortices at the surface and throughout the interior. They are created from mesoscale eddies and strong currents, and they provide a dynamical conduit for energy transfer towards microscale dissipation and diapycnal mixing. Consideration is given to their generation mechanisms, instabilities, life cycles, disruption of approximately diagnostic force balance (e.g. geostrophy), turbulent cascades, internal-wave interactions, and transport and dispersion of materials. At a fundamental level, more questions remain than answers, implicating a programme for further research.

## 1. Introduction

Oceanic submesoscale currents (SMCs) occur on an intermediate scale on the order of 1 km horizontally. This prosaic name is an operational definition developed in relation to mesoscale currents (i.e. eddies), which are well known as the dominant reservoir of kinetic energy in the ocean with a larger horizontal scale of many tens of kilometres [1] and an evolutionary time of weeks. Mesoscale currents have been an active target of research since the 1960s when it first became possible to make sustained measurements of interior currents [2]. For SMCs, their awkward size presents an observational barrier that delayed an appreciation of their abundance: they are large for shipboard instrument detection, small and rapidly evolving for typical ship surveys, small for many satellite remote sensing footprints, and often difficult to distinguish from inertia-gravity waves (IGWs) in single-point time series or individual vertical profiles. In numerical circulation models, the horizontal grid resolution for strong currents and eddies stayed above about 10 km until this century, while more idealized computational simulations of waves and turbulence on smaller scales typically did not include the mesoscale inhomogeneity that spawns SMCs. Theory did not anticipate them well either because their essential dynamics are advective, hence nonlinear and theoretically difficult; i.e. they are a manifestation of turbulence in the regime of marginal control by planetary rotation and stable density stratification. Only in the 2000s with the advent of intermediate-scale simulations on bigger computational grids and the increasing utilization of two-dimensional, high-resolution surface images that reveal organized material patterns, did SMCs become a serious scientific subject, with theory and *in situ* measurements following perforce. Both of these genesis stories are somewhat apocryphal because no doubt many previous observers had noticed the phenomena (vide Charybdis or, more cogently, [3]), but thresholds for widespread awareness are often delayed.

To be more quantitative, the approximate scale ranges for SMCs are ℓ=0.1–10 km in the horizontal, *h*=0.01–1 km in the vertical, and hours-days in time (except for some submesoscale coherent vortices (SCVs) that can wander around in the vertical interior with lifetimes of years). In dynamical terms, ℓ is larger than the turbulent boundary layer thickness *h*_b_, below which the currents are more nearly isotropic and usually non-hydrostatic, and ℓ is smaller than the first baroclinic deformation radius ℓ_*d*_, around and above which the currents are more geostrophic. What makes SMCs dynamically distinct from geostrophic mesoscale currents—a more fundamental distinction than simply their smaller size—is a Rossby number, *Ro*=*V*/*f*ℓ, and Froude number, *Fr*=*V*/*Nh*, that are not asymptotically small. (*V* is a characteristic horizontal velocity scale, *f* is the local Coriolis frequency for Earth’s rotation (assumed spatially uniform as appropriate for the small ℓ of SMCs), and $N=\sqrt{-g\partial_{z}\ln[\rho ]}$ is a density *ρ* stratification frequency, with *z* the upward (anti-gravity) coordinate and *g* the gravitational constant.) Nor are *Ro* and *Fr* asymptotically large, which would move into the realm of isotropic shear turbulence. Commonly *Fr*∼*Ro*, hence there is a strong anisotropy with *h*/ℓ∼*f*/*N*≪1 (as in geostrophic scaling). However, in the vicinity of weakly stratified surface and bottom turbulent boundary layers (SBLs and BBLs), where SMCs are particularly active, *N*(*z*) is highly variable and somewhat ambiguous, so the vertical stratification profile must be considered rather than just a simple scaling number. In fact, the generation and evolution of SMCs is intimately related to the smaller scale turbulence in these boundary layers (§5). The local Richardson number or its vertical profile, *Ri*=*N*^2^/(∂_*z*_*V*)^2^∼*Fr*^−2^, also can assume intermediate values in SMCs. As in most geophysical turbulence regimes, the Reynolds *Re* and Peclet *Pe* numbers are high, so advective dynamics are dominant; *Re*=*V* ℓ/*ν* and *Pe*=*V* ℓ/*κ*, where *ν* and *κ* are molecular diffusivities for momentum and density.

This SMC scale range overlaps to a high degree with IGWs, and the two phenomena must be distinguished by their evolutionary behaviours, with IGWs most evident in their oscillation and propagation. A central question, only partly resolved, is how dynamically interactive SMCs and IGWs are. Often the default answer is that the interaction is weak, but this is not always true (§6f).

This paper gives a perspective on SMC dynamics, i.e. a conceptual framework and a programme for further research. It is by no means a full review of this subject that by now has a large and rapidly expanding literature. It emphasizes theory and modelling more than measurements.

## 2. Roles in the general circulation

**Figure 1.**
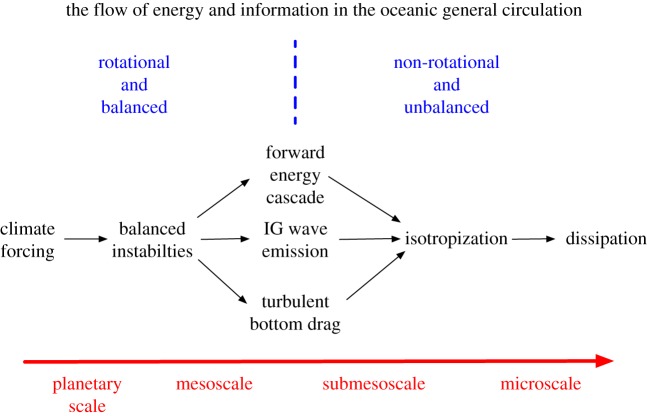
Stages in the oceanic general circulation from planetary-scale forcing to microscale dissipation and mixing. The dynamical parameters *Ro* and *Fr* pass through O(1) values within the submesoscale regime.

The oceanic general circulation and stratification are forced by fluxes of momentum, heat, water and other materials at the sea surface, with the planetary-scale differences the most important.^1^ Climate equilibrium is necessarily achieved through a balancing kinetic energy dissipation rate *ε*, which in a viscous fluid can only occur at a very small-scale, i.e. around centimetres. Thus, there is a dynamical route to dissipation that traverses all the intervening scales (figure 1). From the directly forced large-scale currents, the principal sink is through mesoscale ‘balanced’ instabilities, where balance refers to an approximate diagnostic force balance, either hydrostatic and geostrophic for small *Ro*,

2.1 $$-fv\approx -\partial_{x}\phi ,\;fu\approx -\partial_{y}\phi ,\;\partial_{z}\phi \approx b,$$

or with generalizations that are more accurate at finite *Ro*.^2^ The coordinates **x**=(*x*,*y*,*z*) and velocities **u**=(*u*,*v*,*w*) are aligned with *x* eastward and *z* upward against gravity; where appropriate, a subscript *v* will denote the vertical component of a vector, and *h* will denote its horizontal vector component. *ϕ*=*p*/*ρ*_0_ is pressure normalized by mean density, and *b*=−*g*(*ρ*/*ρ*_0_−1) is the buoyancy anomaly.

Balanced eddies are characterized by an inverse energy cascade (geostrophic turbulence; [4]), so by themselves they do not provide a route to dissipation. As indicated in the diagram, several other classes of motion might help provide the route: (i) BBLs with bottom stress and associated energy loss; (ii) IGWs spontaneously emitted by the eddies or (iii) some other unspecified processes that break the momentum-balance constraints and permit the forward energy cascade towards smaller scales. All of these routes involve smaller scale turbulence that cascades energy down to dissipation. If ℓ and *h* become small enough with a given energy level (*V*) and a rotating, stratified environment (*f*, *N*), both *Ro* and *Fr* become large, and the flow escapes any dynamical inhibition to completing the route to dissipation; furthermore, there is no constraint towards diagnostic momentum-balance in this regime. Overall, the large- and small-scale regimes in figure 1 are relatively well understood, respectively, as geostrophic and non-rotating fluid dynamics. However, the middle regime, with intermediate values of *Ro* and *Fr* at the margin of rotating stratified control, is much more of a frontier, and SMCs cross its border.

The present understanding, from lack of contrary evidence from an abundance of numerical simulations that test the possibility, is that spontaneous emission by mesoscale eddies with small *Ro* and *Fr* is usually weak, although where the bottom flow over topography is strong enough, IGW excitation can be a significant mesoscale energy sink (e.g. within the Antarctic Circumpolar Current; [5,6]). BBL dissipation can also be significant [7]. Nevertheless, most of the ocean’s currents are well separated from the bottom, and there may also be significant eddy energy sinks in the interior and near the surface. This is where SMCs, if they break the balance constraint and exhibit a forward energy cascade, can be a major conduit to dissipation. Some conspicuously large, submesoscale-instigated, upper ocean *ε* values have been reported [8,9], although meaningful global estimates are still lacking.

In oceanic general circulation models (GCMs), even with mesoscale-resolving grids, there is a significant energy sink away from the bottom that is effected through *ad hoc* eddy viscosity parametrizations. This is a necessary ingredient in such models to regularize the grid-scale variance generated by nonlinear advective cascades. It is likely that such eddy viscosities are surrogates for the submesoscale forward cascade processes, though as yet it is unclear how much of their control lies in the mesoscale structures themselves (i.e. could be readily parametrized).

As an aside, there is also considerable oceanic kinetic energy in the lunisolar tides and surface waves, but their generation and routes to dissipation are largely separate from the general circulation’s, and dynamically they are rather loosely coupled to SMCs as well (§§6f,g).

Apart from this global energy perspective, there are other important system-wide effects provided by SMCs:

A central problem in meteorological and other geophysical forecasts is model initialization from measurements. An important ingredient is constraining the model fields to be approximately momentum-balanced and thus express an evolution on a hypothesized ‘slow manifold’ (i.e. without any IGWs). However, implementation of balanced initialization schemes in numerical weather prediction models have often failed to converge, with the implication of balance breakdown as a generic phenomenon [10] that is a hallmark of SMC dynamics.

The global overturning (thermohaline) circulation brings dense water upward through the pycnocline, which can only occur if the diapycnal (i.e. across density surfaces, effectively vertical) material eddy diffusivity *κ*_v_ is large enough to allow its transformation to lighter water. The common view is that IGW dissipation, often through wave breaking, is the dominant cause of *κ*_v_, but if there is a significant forward energy cascade by SMCs, then it may also contribute appreciably to larger *κ*_v_∼*ε*/*N*^2^ values.^3^ This seems especially plausible near the surface and bottom, but it is a serious possibility even in the interior (§6d).

**Figure 2.**
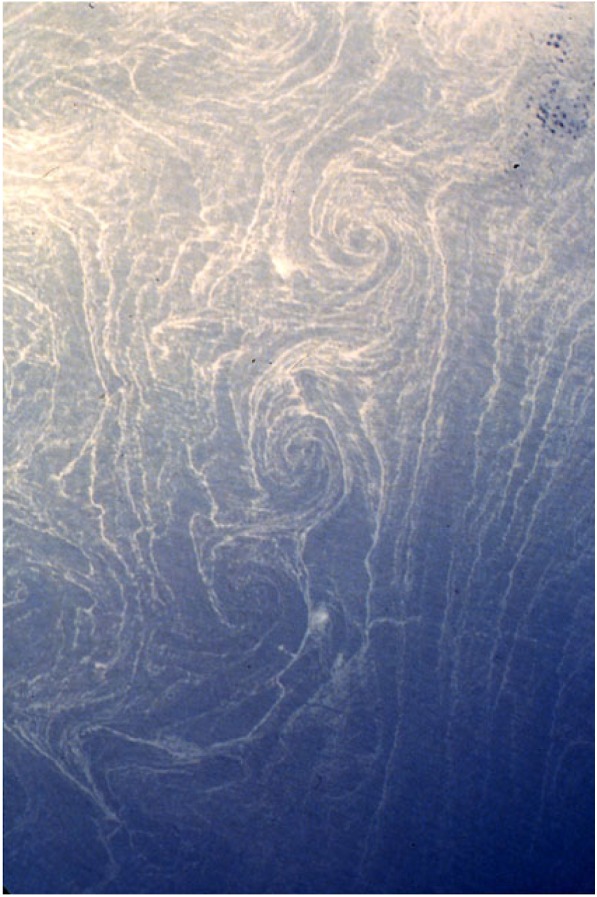
Sun-glintshowing ‘spirals on the sea’ not far off the Mediterranean coast of Africa photographed from space [19,20]. The vortex radii are ≈5 km and the high surfactant concentration *c* occurs in convergence lines that are hundreds of metres wide, probably due to dense filament arms in a spiral configuration inside the vortices. The pattern suggests a vortex-street roll-up has occurred from a lateral shear instability of some parent front, filament or topographic wake.

SMCs have a large vertical velocity *w*, especially within the SBL, much larger than for mesoscale eddies and with larger space and time scales than for boundary layer turbulence. This leads to large material eddy fluxes, $\bar{w^{\prime }c^{\prime }}$, by SMCs. (*c* is a material concentration that is passively advected and mixed by the fluid dynamics; the overbar denotes an average; and the prime denotes a fluctuation about the average.) In particular, SMCs manifest both ‘mixed-layer instability’ (i.e. baroclinic instability of a weakly stratified layer in the presence of a horizontal buoyancy gradient) [12] and frontogenesis [13], and both of these processes have $\bar{w^{\prime }b^{\prime }}>0$. This is an essentially adiabatic restratification flux that opposes the diabatic destratification flux (mixing) by boundary layer turbulence. It is also a conversion of potential energy to kinetic energy, and thus is indicative of one important process for SMC generation (§§5a,b). When *c*=*n* is a biological nutrient, then $\bar{w^{\prime }n^{\prime }}>0$ can be an important fuel for plankton productivity in the euphotic zone [14–18]. The surface convergence lines lie above *w*<0 downwelling sheets. They trap biogenic surfactants *c* that suppress short surface gravity waves and allow a visualization of SMCs at the surface through spatial modulation of sunlight and radar reflections (figure 2).

**Figure 3.**
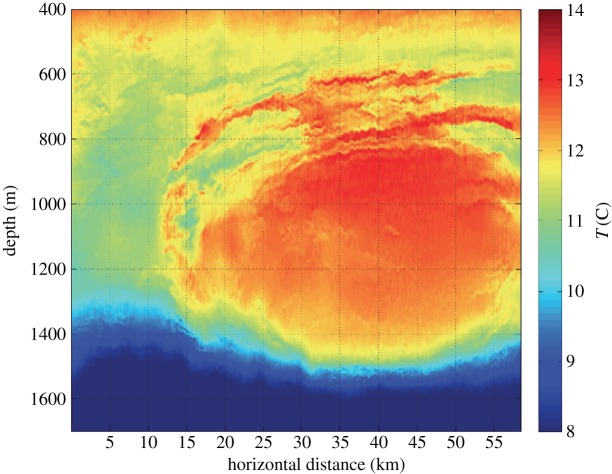
Acoustic image of a temperature cross-section in a Meddy SCV (i.e. SMC eddy of Mediterranean origin) after detachment from the Iberian abyssal boundary current [21]. The SCV is approximately axisymmetric around a central vertical axis. The warm temperature anomaly is accompanied by a positive salinity anomaly such that the vortex centre is neutrally buoyant with a local minimum in |∂_*z*_*ρ*|. The *T*/*S* anomaly implicates an SCV origin in the subsurface outflow of dense Mediterranean water into the Atlantic, where it descends as an entraining gravity current to a level of neutral buoyancy and flows poleward along isobaths. The encircling anticyclonic circulation is in gradient-wind, hydrostatic momentum-balance with the buoyancy field. The maximum azimuthal velocity is at *z*≈−1100 m. Meddies are agents in the lateral spreading of the *T*/*S* water mass throughout the subtropical Atlantic [22].

Perhaps the earliest known class of SMCs is the so-called SCVs (figure 3). They were detected as spatially sparse but abundant instances of an extreme chemical anomaly *c*′ in hydrographic profiles in association with a local interior minimum in vertical stratification |∂_*z*_*ρ*|. The interpretation, often later confirmed by velocity measurements, is that these are gradient-wind balanced anticyclonic vortices that trap their core water materials and live long enough (years) to be advected far away from their generation site at which the encapsulated *c* value is ordinary; thus, SCVs have a long-range transport capacity for dissolved materials. From measurements, it is clear that SCVs are common throughout the interior of the ocean and contain distinctive *c*′ suites that, from the persistent chemical geography in the ocean (i.e. water masses), indicate different origin locations. The dominance of anticyclonic SCVs over cyclonic ones was originally interpreted as due to local diapycnal mixing events that create a stratification anomaly followed by gradient-wind adjustment of the vortical flow [23,24]. A more refined interpretation is that SCVs usually form from separating, violently unstable boundary currents that induce strong mixing and roll up into vortices [25] (§5d).

Thus, SMCs are an important element in the general circulation from several perspectives. In retrospect their existence might have been inferred from the roles they play, though this was not the earlier public discourse. Notice the substantial degree of hypotheticality in these characterizations; there is still much to quantify about SMC contributions to oceanic system dynamics.

## 3. Modelling methodologies

**Figure 4.**
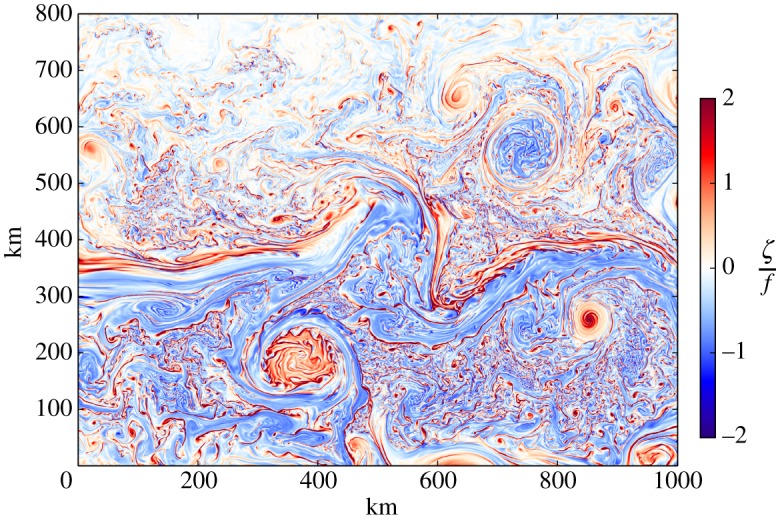
Vertical vorticity *ζ* normalized by *f* at the surface in the wintertime Gulf Stream after separation from the western boundary in a nested-subdomain simulation. Notice the meandering Stream in the centre, the northern warm anticyclonic and southern cold cyclonic mesoscale Rings, and the nearly ubiquitous submesoscale features of many types: North Wall ‘comma’ instabilities and streamers, Ring interior instabilities, cyclonic coherent vortices, and ‘submesoscale soup’ in-between. Notice the asymmetry between the red and blue colours: cyclonic vorticity is typically stronger in the surface layer, due to finite-*Ro* effects of vortex stretching in (5.4) and ageostrophic instabilities (§6a) and loss of balance (§6d) that limit the anticyclonic vorticity amplitude to ζ/f≳−1. The colour bar does not fully span the field range; i.e. there are many places with *ζ*/*f*>2 [27].

Because of the barriers for SMC measurement and theory (§1), modelling has led the discovery of submesoscale phenomena. There is a long and fruitful practice in geophysical fluid dynamics, and in computational fluid dynamics more generally, of isolating and idealizing a phenomenon to simulate it. An example of this is large eddy simulation (LES) as applied to a turbulent boundary layer, usually with assumptions of homogeneity (expressed as horizontal periodicity) if not also isotropy and stationarity; LES uses turbulent mixing parametrizations only for the finest spatial scales with h,ℓ≲1 m. This idealized approach can also be practiced for SMCs, and it has been done usefully in many instances (e.g. the instability of a surface front [26]). However, the origins of SMCs are mainly from mesoscale inhomogeneities, and an alternative approach of multiply nesting subdomains with successive refinements of the grid size provides a powerful depiction of their spontaneous emergence and subsequent evolution in the context of the mesoscale environment, which in turn is usually dependent on the basin-scale circulation (figure 4). For realistic simulations, the technique is to simulate the circulation on at least the scale of a whole ocean basin with whatever climatological boundary conditions are required, then choose a subdomain of interest and use open boundary conditions taken from the solution in the larger domain. This is algorithmically delicate, but satisfactory methods have been developed [28]. Experience shows that the subdomain should be large enough to develop its own intrinsic variability on the newly available scales, and that the grid refinement factor should not be much larger than 3. This nesting step can be repeated any number of times, up to the limits of computer time, scientific quality checking and model validity (for most circulation models that solve the hydrostatic Primitive Equations, this is associated with non-hydrostatic dynamics, whose transition seems to occur in most SMC phenomena around a horizontal grid size of tens of metres). Once the horizontal grid size is around 1 km or less—i.e. adequate for both the surface deformation radius, ℓ_s_∼*N*_s_*h*_b_/*f*, where *N*_s_ is the stratification frequency within the weakly stratified SBL, and the width of boundary-slope currents ℓ_b_; §5 and figure 7)—experience shows that SMCs spontaneously emerge in many if not most situations. Again experience shows that the most evident benefits of multi-step nesting accrue from one-way downscaling, consistent with the depicted flow of dynamical information from larger scales to smaller ones in figure 1. Two-way nesting is feasible, though at a computational price, and it is as yet infrequently practiced. Another advantage of realistic, nested-grid simulations is that they offer more opportunities for discovery of new phenomena (i.e. surprises) than do idealized models configured on the basis of *a priori* expectations.

## 4. Conceptual frameworks

### (a) Coherent structures

As in many other kinds of turbulence, SMCs exhibit recurrent patterns that represent preferred slowly or self-similarly evolving states. The two basic paradigms are parallel flow (*v*(*x*,*z*) with ∂_*y*_=0) or axisymmetric flow (*V* (*r*,*z*) with ∂_*θ*_=0). In a rotating, stably stratified fluid in the absence of non-conservative forces or other adjacent flows, both patterns are steady-state configurations in horizontal geostrophic or gradient-wind and vertical hydrostatic momentum-balances with the pressure and buoyancy fields. Parallel flow is an idealized template for an SMC front or filament, where the former has a single-sign horizontal buoyancy gradient and the latter a central buoyancy extremum. Axisymmetric flow is a template for an SMC vortex. To be categorized as an SMC (distinguished from mesoscale), the front or filament has to have a cross-axis scale less than 10 km or so, and the vortex has to have a radius similarly bounded; a lower size limit (distinguished from microscale) would be where rotational dynamics are irrelevant, *Ro*≫1.

These template patterns are thus ‘safe harbours’ sheltered from rapid advective deformation. More importantly, they are advective attractor states in the sense that asymmetric perturbations tend to be stretched out in the symmetry direction and transfer their energy into the symmetric current [29,30], as long as the latter has a stable shear profile. This behaviour allows the templates to act as coherent structures with relatively long lifetimes limited only by destructive encounters with other strong flows or by non-conservative forces, e.g. by boundary fluxes and boundary-layer turbulence. The latter gives rise to a usually weaker, ageostrophic secondary circulation in the plane perpendicular to the symmetry direction, which in turn is an agent for further evolution of the coherent structure. SMC fronts, filaments and vortices arise most efficiently near the top and bottom boundaries (§5). Broadly speaking, the surface SMC structures are different in shape and behaviour among different mesoscale environments: strong currents, strong eddy interiors (e.g. inside Rings) and the more amorphously patterned regions elsewhere (i.e. the SMC soup; figure 4). Nearer the bottom SCVs arise in association with the separation of currents from topography with an active BBL influence (i.e. vortical wakes).

### (b) Momentum-balance

In a rotating, stratified fluid with small *Ro* and *Fr* and large *Re*, the reigning dynamical framework is Quasigeostrophy (QG), which has yielded many successful theoretical explanations of large- and mesoscale phenomena in the ocean and atmosphere. Its solutions manifest geostrophic turbulence, form three-dimensional coherent vortices with distinctive patterns in QG potential vorticity *q*_*qg*_, and generate filaments in *q*_*qg*_ and other material tracer fields that are horizontally advected in a dynamically passive way by the geostrophic velocity **u**_*g*_ from stronger adjacent eddies (e.g. in the forward inertial cascade of potential enstrophy $\bar{q_{qg}^{2}}$; §6d) [31]. QG has a subclass especially relevant to near-boundary regions, Surface QG (SQG), based on an idealization of *q*_*qg*_(**x**) that is zero in the fluid interior, so its evolution is controlled by a horizontal advection–diffusion equation for horizontal buoyancy with gradients ∇_h_*b* on the boundary^4^ [32,33].

**Figure 5.**
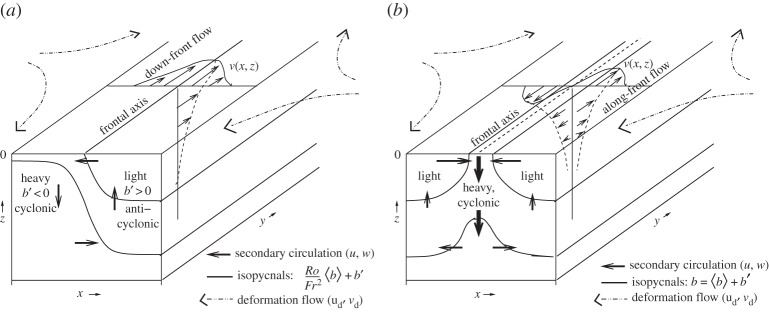
Sketches of surface-layer frontogenesis caused by a large-scale deformation flow for front **u**_d_ (*a*) and dense filament (*b*) configuration in *b*(*x*,*z*). The along-axis flow is partly geostrophic, and the secondary circulation in the (*x*,*z*)-plane is ageostrophic. With time the *x* gradients sharpen at a super-exponential rate, and *Ro* grows. With finite *Ro*, the downwelling *w*<0 and cyclonic *ζ*/*f*>0 features dominate their opposite-sign counterparts.

When approaching SMC behaviours, QG theory should be a starting point; e.g. the shapes of the currents in figure 5 are roughly in conformity with a QG frontogenesis solution in the presence of a deformation flow **u**_d_. Yet, as noted in §1, the *Ro* values for SMCs are usually not small, so QG should not be expected to be quantitatively accurate, and in some instances (e.g. the forward cascade of total energy) it is even qualitatively wrong. The next step on a theoretical path should be nonlinear Balance Equations (BE) models with higher order accuracy asymptotically in *Ro*≪1. Many BE models have been devised by generalizing the momentum-balance constraint while still neglecting ageostrophic acceleration, ∂_*t*_**u**_*a*_, and often they have provided accurate explanations [34] (e.g. the gradient-wind relationship between *V* (*r*,*z*) and *b*(*r*,*z*) in a Meddy; figure 3). Although less explored than QG and without its simple inertial range theory (§6d), experience indicates that BE solutions also exhibit a mostly inverse energy cascade behaviour [35]. Nevertheless, even BE models often fail to have solutions for large *Ro*; most computational BE solution methods are iterative, and the common manifestation of this failure is iteration non-convergence (N.B. the first bullet in §2; also see §6a). In more general fluid dynamics, an evolutionary approach to a point of BE failure would, by definition, exhibit unbalanced dynamical behaviour, whether as spontaneous emission of IGWs or, as seems more common, as a spontaneous imbalance feeding into a forward energy cascade. Even for somewhat larger *Ro* values than where BE solutions are known to converge, simulations indicate that SMC solutions often remain close to satisfying momentum-balance relations [36,37]. This evolutionary closeness to balance, sometimes called the fuzziness of the slow manifold, still needs further theoretical explication.

A measured and simulated property of SMC turbulence in the SBL is a horizontal wavenumber spectrum *E*(*k*_h_) of velocity (or buoyancy) that is shallower than the *k*^−3^_h_ shape of a QG potential enstrophy inertial cascade range [38,39]. With a scaling estimate for the velocity amplitude at wavenumber *k*_h_, $V_{k}∼{\left(k_{h}E\right)}^{1/2}\gg k_{h}^{-1}$, the wavenumber-dependent $Ro_{k}=k_{h}V_{k}/f∼{\left(k_{h}^{3}E\right)}^{1/2}\gg k_{h}^{0}$ will grow with increasing *k*_h_ for a shallower *E*≫ *k*^−3^_h_ shape; i.e. in such cases, SMC turbulence is increasingly inconsistent with QG or BE models at smaller scales (§6d).

On the other hand, the widespread experience is that many flows are not far from satisfying momentum-balance relations, indicating that strong balance breakdowns may often be local and transient, followed by an approximate restoration of balance for whatever remains after forward cascade to dissipation or IGW radiation. This conjecture does not yet have a firm theoretical proof.

## 5. Generation

As described in §3, SMCs spontaneously arise in simulations of mesoscale-active flows when the grid scale is fine enough and *Re* is large enough. In some generic sense, this implicates mesoscale instability as their origin, but several particular processes seem most relevant. The ones discussed in this section are geographically generic (i.e. in the SMC soup in the SBL in contrast to special flow structures that arise within strong, laterally sheared currents like the Gulf Stream or its Rings (figure 4) or near extreme topographic shapes). Furthermore, the SBL and BBL are featured here as the primary generation sites, but interior generation is also a viable process (§6d).

### (a) Mixed-layer instability

The simplest conception of surface-layer SMCs is baroclinic instability within this weakly stratified layer (due to efficient vertical buoyancy mixing by boundary layer turbulence), with a horizontal buoyancy gradient $\nabla_{h}\bar{b}$ along the boundary and a more strongly stratified interior *N*(*z*) below. This is in contrast to the baroclinic instability centred within the pycnocline that is understood to be a primary generating process for mesoscale currents with horizontal scales around the baroclinic deformation radius ℓ_d_. A weakly stratified surface layer has its own deformation radius, ℓ_s_∼*N*_s_*h*_b_/*f*, which is typically much smaller than ℓ_d_ because of the relative smallness of its stratification *N*_s_ and thickness *h*_b_; e.g. for *N*_s_=10^−3^ s^−1^, *h*_b_=10^2^ m and *f*=10^−4^ s^−1^, ℓ_s_=1 km. The horizontal length scale of the most unstable linear mode is ≈ℓ_s_, so this instability generates fluctuations within the submesoscale range. Satellite images show an abundance of sea-surface temperature gradients, and the surface layer is often close to neutrally stratified, hence the conditions for mixed-layer instability (MLI) are commonly satisfied.

Baroclinic instability associated with a boundary buoyancy gradient $\partial_{x}\bar{b}$ has deep roots in the QG theories of Charney [40] and Eady [41], devised as an explanation of extratropical atmospheric cyclogenesis. In the SMC context, the dependence of linear instability on ℓ_s_∝*N*_s_ is problematic because the latter is highly variable due to intermittent turbulent mixing in the SBL. Similarly, the unstable geostrophic-mode mode growth rate, $\sigma_{mli}∼\partial_{x}\bar{b}/N_{s}=f\;\partial_{z}\bar{v}/N_{s}$, is ill-determined for the uncertain and variable *N*_s_ in the SBL. Beyond the onset of linear instability, however, MLI exhibits a finite-amplitude regime in idealized simulations with a $\partial_{x}\bar{b}(x,z)\neq 0$ within a surface layer above a stronger interior stratification whose quasi-equilibrium phase is independent of the initial *N*_s_ and even creates its own *N*_s_>0 value in the equilibration phase due to restratification flux, $\bar{w^{\prime }b^{\prime }}>0$ (§2; [12,42]).

A useful characterization of this regime and its buoyancy flux is in terms of its eddy-induced transport velocity u*. This is an increment over the Eulerian-averaged velocity that adds to its advection of averaged material concentrations, $\nabla \cdot \bar{{\text{u}}^{\prime }c^{\prime }}\approx {\text{u}}^{*}\cdot \nabla \bar{c}$, where the average is made over the eddy fluctuations [43], here for SMC eddies. For a two-dimensional configuration in $\bar{b}(x,z)$, a mean eddy-induced overturning (or secondary circulation) velocity is defined by

5.1 $$u^{*}=-\partial_{z}\Phi^{*},\;v^{*}=0,\;w^{*}=\partial_{x}\Phi^{*},$$

where the MLI overturning streamfunction is defined by

5.2 $$\Phi^{*}∼-\frac{h_{b}^{2}\;\partial_{x}\bar{b}(x,0)}{f}$$

(assuming *f*>0 in the Northern Hemisphere), with a simple convex shape in *z* across the surface layer and a non-dimensional coefficient determined by fitting it to simulation results [12]. Note that there is no dependence on $N^{2}=\partial_{z}\bar{b}$. u* is an ageostrophic current because it is not in balance with the pressure gradient force. In an unstable frontal region with $\partial_{x}\bar{b}(x,0)>0$ over a finite extent in *x* (e.g. in between two mesoscale eddies), the u* circulation is an overturning cell, upward on the lighter side in $\bar{b}$ and downward on the denser side, with connecting opposite-sign horizontal flows in the upper and lower parts of the surface layer. The corresponding vertical buoyancy flux is

5.3 $$\bar{w^{\prime }b^{\prime }}∼-\Phi^{*}\partial_{x}\bar{b}∼\frac{h_{b}^{2}{\left(\partial_{x}\bar{b}(x,0)\right)}^{2}}{f}>0$$

[12,44], with an analogous expression for $\bar{u^{\prime }b^{\prime }}$.

Eddy-induced advection is widely used as a basis for eddy parametrization in GCMs, mainly to represent mesoscale effects, and it is also apt for MLI eddies. It expresses a sink of mean available potential energy into eddy energy, and it acts as an eddy-induced advection that tilts sloping mean isopycnal surfaces towards the horizontal, which has the effect of increasing the mean stratification. On the face of it, this $\bar{w^{\prime }b^{\prime }}>0$ is an up-gradient eddy flux in the presence of $N^{2}=\partial_{z}\bar{b}>0$ (i.e. implying a negative eddy diffusivity in *z*), but this is a misleading way to view the baroclinic instability process whose flux is essentially adiabatic rather than diapycnal. Other eddy-averaged material concentrations besides *b* do experience eddy-induced advection along with an eddy diffusion process that is fully three dimensions in the surface layer and oriented along isopycnal surfaces in the interior. Because of the large *w* in the SBL one can expect a down-gradient (i.e. mixing) material flux $\bar{w^{\prime }c^{\prime }}$ in the presence of a well-defined mean gradient $\partial_{z}\bar{c}(z)$ [45], in concert with the down-gradient flux by SBL turbulence.

In the finite-amplitude, quasi-equilibrium phase of idealized MLI simulations, the submesoscale eddy field develops sharp frontal features (i.e. strong gradients) that have an along-front size close to the eddy scale [12]. In this regime, the phenomenon is also called mixed-layer eddies (MLEs). Their frontal structures are an indication of the finite *Ro* values typical of SMCs as well as of the manifestation of frontogenesis within the eddies.

A rationale for the scaling relationship for Φ*, hence also for $\bar{w^{\prime }b^{\prime }}$, is that the energy reservoir that sustains MLEs is the available potential energy density in the surface layer $∼h_{b}^{2}|\nabla_{h}\bar{b}|$. This reservoir is also relevant to strain- and TTW-induced frontogenesis (§5b,c).

### (b) Strain-induced frontogenesis

**Figure 6.**
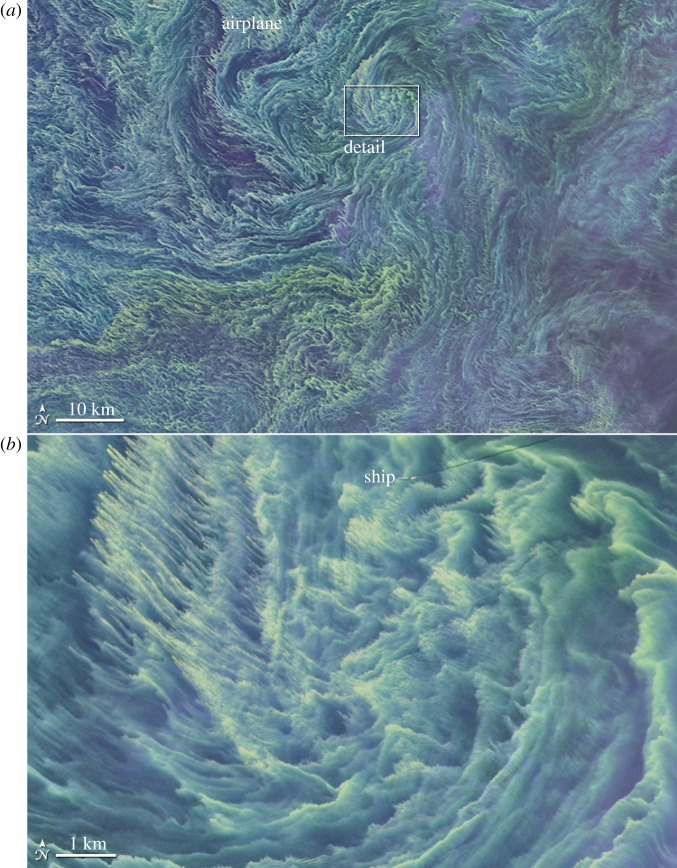
Landsat 8 false colour image of a large bloom of cyanobacteria in the Baltic Sea on 11 August 2015. Panel (*b*) is a zoom of the indicated box region in (*a*). The reference length bars are 1 km and 10 km, respectively. The concentration patterning is by surface convergence lines in the soup of submesoscale fronts and filaments, plus perhaps some SBL Langmuir circulations in the upper-left corner of the zoom. See http://landsat.visibleearth.nasa.gov/view.php?id=86449.

Surface vertical vorticity, $\zeta =\hat{\text{z}}\cdot \nabla \times \text{u}=\partial_{x}v-\partial_{y}u$ (figure 4), sea-surface temperature and material concentration *c* (figures 2 and 6) patterns typically show linear (horizontally elongated) submesoscale features. These are signs of the effect of frontogenesis that makes sharp edges (buoyancy fronts) or line patterns (buoyancy filaments or high *c* values for surfactants drawn into horizontal convergence lines). A paradigm for generating such patterns is frontogenesis [13]. This is a classical dynamical process in meteorology, whereby a favourably aligned surface horizontal buoyancy gradient in a background horizontal deformation flow with a uniform strain rate (e.g. *u*_d_=−*αx*, *v*_d_=*αy*, *w*_d_=0) has a rapidly growing magnitude. For a passive tracer *c* in **u**_d_, the rate of growth is exponential, $\partial_{x}c∼\exp[\alpha t]$ and for the dynamically active *b* with finite *Ro*, the rate is super-exponential. With a momentum-balance assumption, ∂_*x*_*b* even has a finite-time singularity [46]; however, rather than a prize-winning proof of a singularity in fluid dynamics,^5^ this is better interpreted as an evolution that will force a breakdown of momentum-balance, either as spontaneous emission or spontaneous imbalance. Nevertheless, frontogenesis is an extremely efficient way to transfer variance and energy density to smaller scales. In the atmosphere, fronts are relatively rare at a given time because only a few parent synoptic circulations can fit within Earth’s surface area, whereas in the ocean their scale is much smaller and they are much more abundant (figure 6).

In the context of SMCs, the primary background strain is from mesoscale currents and eddies, and the seed buoyancy gradients are created either by QG chaotic advection of the mesoscale *b* field or by a process like MLI that has amplifying submesoscale *b* fluctuations. The flow configurations of strain-induced frontogenesis are sketched in figure 5 for both a buoyancy front and dense filament at the surface. The buoyancy gradient ∂_*x*_*b*(*x*,*z*) is surface intensified or at least strongest in the weakly stratified surface layer. It has an associated, approximately geostrophic shear along the frontal axis, *f*∂_*z*_*v*_*g*_=∂_*x*_*b* and *v*_*g*_(*x*,*z*) itself is strongest at the surface. For a front *v*_*g*_ is a jet, and for a filament *v*_*g*_ is a pair of opposite-sign jets.

In the cross-frontal plane is an ageostrophic, O(Ro) secondary circulation (*u*,*w*) forced by the deformation flow. For a front, the circulation is a single overturning cell with upwelling and surface divergence on the light side and downwelling and surface convergence on the dense side. The vertical motion *w* induces additional vertical vorticity *ζ* (i.e. beyond the *ζ*_*g*_ component) by vertical vortex stretching, where

5.4 $$\partial_{t}\zeta \approx (\;f+\zeta )\partial_{z}w+\cdots .$$

In the upper part of the layer (where ∂_*z*_*w*<0 is largest in *z*) on the light side of the front, vortex stretching generates anticyclonic vorticity, and *vice versa* on the dense side. The second right-side factor is an O(Ro) effect (i.e. beyond QG), and with the finite *Ro* in SMCs, it combines with the first factor to amplify the cyclonic vorticity more than it does the anticyclonic vorticity. Thus, the surface vorticity field develops a strong cyclonic skewness (N.B. a prevalence of the red colour at the smaller scales in figure 4). Through an O(Ro) advective feedback on the secondary circulation, the frontogenetic *w* field develops a strong negative skewness with dense-side downwelling stronger than light-side upwelling. And through an O(Ro) advective feedback in the buoyancy equation, the secondary circulation enhances the frontogenetic rate beyond that due to *α*, thus leading to the super-exponential growth in ∂_*x*_*b*.

In a dense filament, there are two secondary circulation cells that come together in a central downwelling branch underneath a surface convergence line, which, as above, induces cyclonic vorticity there, with again large skewness values for *w* and *ζ*. The central convergence in this secondary circulation provides an even more effective O(Ro) advective feedback in the buoyancy equation, hence a more rapid growth in ∂_*x*_*b* than in a front of comparable size and strength [47]. Frontogenesis can also occur for a light surface filament (i.e. by reversing all the signs in the dense filament sketch), but in this configuration the secondary circulation has a surface divergence in the centre that is frontolytic in opposition to the strain-induced frontogenesis. Thus, light filament frontogenesis is inherently weaker and seemingly much rarer in nature.

If only strain-induced frontogenesis is occurring, then the secondary circulations and their associated vertical buoyancy flux (averaged over the circulation cell) are

5.5 $$u=-\partial_{z}\Phi ,\;w=\partial_{x}\Phi ,\;\Phi ∼-\frac{\alpha h_{b}^{2}\partial_{x}b}{f^{2}}\;\mathrm{and}\;wb∼-\Phi \;\partial_{x}b∼\frac{\alpha h_{b}^{2}{\left(\partial_{x}b\right)}^{2}}{f^{2}}>0,$$

where *Φ* is the overturning streamfunction for the ageostrophic circulation.^6^ The similarities with (5.1))–(5.3) are striking. Yet here the interpretation is rather different. This is the secondary circulation and its buoyancy flux for a single frontal feature forced by *α*>0, not the eddy-induced circulation due to a field of MLEs. Yet both processes have their energy source in the background available potential energy in the surface layer, and both have a restratification buoyancy flux. The total vertical buoyancy flux in a field full of frontal features is obtained by summing up all their individual contributions in (5.5) (assuming mutual interactions do not interfere with their behaviour in isolation).

Both dense filaments and fronts are commonly observed in both nature and simulations, as well as hybrid forms. Because both shapes can undergo strain-induced frontogenesis, their relative abundance is likely to be determined by the prevalent shapes in the seed population for ∂_*x*_*b*. It is easy to imagine a blending of MLI and frontogenesis in generating surface-layer SMCs. The first process identifies an energy source, and the second process characterizes the advective evolution towards frontal and filamentary lines, as is also seen in MLEs. This is somewhat analogous to atmospheric extratropical cyclongenesis, where baroclinic instability characterizes the early growth of synoptic-scale fluctuations and mesoscale frontogenesis ensues from the advective interaction of the synoptic-scale deformation flow with its near-surface buoyancy gradients [48].

### (c) Turbulent thermal wind

Both the MLI and strain-induced frontogenesis generation processes are formulated within a conservative flow paradigm, yet the oceanic surface layer is almost always turbulent, hence non-conservative. Furthermore, in analyses of surface-layer fronts and filaments in complex-flow simulations, it is often difficult to identify either the parent flow that might be the source of MLI or the sometimes brief intervals of strain-induced frontogenesis with its relevant local strain rate *α*. What does seem common in such structures is an approximate linear momentum-balance called turbulent thermal wind (TTW) plus an incompressible mass balance:

5.6 $$\left.\begin{array}{rl} -\;fv & =-\partial_{x}\phi +\partial_{z}(\nu_{v}\partial_{z}u),\\ fu & =-\partial_{y}\phi +\partial_{z}(\nu_{v}\partial_{z}v)\\ \;\text{and}\;\partial_{z}\phi & =b,\;\nabla \cdot \text{u}=0, \end{array}\right\}$$

with surface boundary conditions of *w*≈0 and $\nu_{v}\partial_{z}\text{u}\to \tau_{s}/\rho_{0}$, and interior boundary conditions of $\partial_{z}\text{u},\partial_{x}b\to 0$ [36]. ***τ***_s_ is the surface wind stress, and *ν*_v_(*x*,*z*) is the vertical eddy viscosity associated with SBL turbulence as determined from some parametrization model.^7^ TTW is simply a composite generalization of geostrophic, hydrostatic (thermal wind) balance, on the one hand, and Ekman boundary-layer balance on the other. When $\hat{\text{z}}\times \nabla_{h}b$ is non-zero and *ν*_v_ is large within the SBL, these two types of circulation are coupled, and an ageostrophic secondary circulation arises around the submesoscale structure that would not be present in either of the simpler balances alone (assuming uniform ***τ***_s_). This system can be viewed as a diagnostic one for **u**_*a*_ for specified *b* (hence *ϕ* and **u**_*g*_), *ν*_v_ and ***τ***_s_ fields. A simple rule of thumb is that **u**_*a*_ has a component near the surface opposing the geostrophic shear **u**_*g*_ in the surface boundary condition and vertical mixing terms, which implies that **u**_*a*_(*x*,*y*,0) will be oriented both to the rear and to the left side (with *f*>0) relative to **u**_*g*_(*x*,*y*,0) with a comparable magnitude, and in the lower part of the SBL **u**_*a*_ will reverse its direction. In two-dimensional *b*(*x*,*z*) configurations as in figure 5, this rule implies that the (*u*,*w*) shapes (apart from a wind-driven Ekman component for *u*) will have the same shapes in TTW as they do in *α*-induced frontogenesis. This further implies that frontogenesis can occur because of TTW in association with the surface convergence lines on the dense side of a front and in the centre of a filament. This evolution is confirmed in [37] for a dense filament with parametrized *ν*_v_, and, if TTW balance is assumed to hold exactly, a finite-time singularity is also predicted in ∂_*x*_*u* and ∂_*x*_*v* at the filament centre, indicating again both the efficiency of frontogenesis in scale shrinkage and the likelihood of an evolutionary breakdown of momentum-balance.

For TTW, the secondary circulation overturning streamfunction and vertical buoyancy flux exhibit the following scalings [49]:

5.7 $$\Phi ∼-\frac{\nu_{v}\partial_{x}b}{f^{2}}∼-\frac{u_{*}h_{b}\;\partial_{x}b}{f^{2}}\;\mathrm{and}\;wb∼-\Phi \;\partial_{x}b∼\frac{u_{*}h_{b}\;{\left(\partial_{x}b\right)}^{2}}{f^{2}}>0.$$

The alternative forms for *Φ* are based on the scaling for the eddy viscosity in a wind-driven turbulent boundary-layer, *ν*_v_∼u_*_h_b_, where $u_{*}=\sqrt{\tau_{s}/\rho_{0}}$. What is of primary importance for *Φ* in TTW is *ν*_v_≠0, and ***τ***_s_≠0 is merely one means (albeit a common one) of maintaining boundary-layer turbulence. The relations in (5.7) are also closely analogous to (5.2), (5.3) and (5.5). All of these processes are characterized by submesoscale generation from conversion of surface-layer available potential energy to kinetic energy, *wb*>0, and its implied restratification flux, whether the energy source is from the mesoscale or from within the proto-fronts and filaments themselves in a further downscale flux among SMCs.

Thus, there is a unified commonality of SMC behaviours in the SBL, whether arising from MLI → MLEs or from frontogenesis induced by *α* and *ν*_v_; no doubt many combinations of these generation processes occur in nature. They have in common stronger secondary circulation and restratification flux with increasing SBL buoyancy gradient ∇_h_*b* and SBL depth *h*_b_ and with decreasing latitude, 1/*f*; in addition, they have increasing strength with ambient strain rate *α* and SBL mixing intensity *ν*_v_. In particular, winter cooling and storminess (i.e. larger *h*_b_ and *ν*_v_) favour stronger SMC activity [50].^8^ Surface SMCs are mainly confined to the SBL for several reasons: MLI depends on the small *N*_s_ there, TTW depends on the large *ν*_v_ there, and the penetration depth into the underlying pycnocline is short (e.g. the Prandtl depth *h*_*P*_∼*f*ℓ/*N*_*pyc*_≈20 m for *f*=10^−4^ s^−1^, ℓ=1 km, and *N*_*pyc*_=5×10^−3^ s^−1^). Accompanying these generation and maintenance processes, of course, are all the destruction processes for fronts and filaments, whether frontal arrest, fragmentation by frontal instability and vortex formation, weakening by SBL turbulent diffusion, or other avenues of forward energy cascade (§6).

### (d) Topographic wakes

Wakes commonly occur in fluid dynamics (e.g. horizontal flow past a vertical cylinder). When *Re* is large enough, wakes exhibit vertical vorticity generation at the boundary in a thin, turbulent boundary layer; horizontal flow separation from the boundary that carries a zone with high lateral shear into the interior; instability of their high lateral shear; amplification of these fluctuations and finite-amplitude roll-up into sometimes long-lived vortices (e.g. in a vortex street; cf. figure 2); and strong lateral mixing of material concentration *c* gradients across their width by the vortical flow field. The same phenomenological sequence occurs in the ocean, but with several important dynamical differences: rotation and stratification are significant (*Ro* and *Fr* are not large); the wakes and their evolution are more fully three-dimensional due to non-uniformity in *z* of both the incoming flows and the boundary shape; and the boundary is essentially only a bottom and not a side (coastal cliffs a trivial exception), which means that the vorticity-generating boundary layer is the BBL. Even in an idealized rotating, stratified simulation with unrealistic side walls, the lateral boundary layer and wake shear layers are quite thin when *Re* is large (or, more precisely, the horizontal Ekman number, *Ek*_h_=*ν*_h_/*f*ℓ^2^, is small), so the phenomenon can be viewed as usually within the submesoscale range [51].

**Figure 7.**
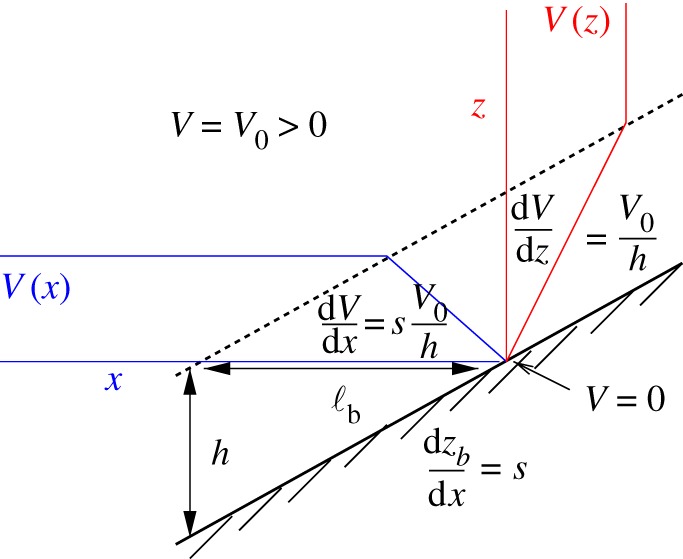
Sketch of the along-slope velocity structure *V* (*x*,*z*) for a uniform interior flow and a turbulent BBL over a bottom slope with *s*=∂_*x*_*z*>0. With a uniform interior *V*_0_, the surface drag generates a vertical shear profile ∂_*z*_*V* (*z*)>0 within the BBL that also projects as a horizontal shear layer with vertical vorticity *ζ*(*x*)=∂_*x*_*V* (*x*)<0 and width ℓ_b_∼*h*_b_/*s*. This vorticity injection sets up a wake instability if there is a subsequent current separation from the boundary [53].

For horizontal flow over a flat bottom, the Ekman BBL with a bottom drag stress ***τ***_b_ generates vertical shear *V* (*z*) within a thin layer of thickness *h*_b_ typically tens of m. This flow only has horizontal vorticity, and there is little likelihood of current separation due to the suppression of vertical motions by *f* and *N*, so the primary instabilities and turbulence are within the BBL itself. With a sloping bottom, other possibilities arise. One is the current-induced generation of lee IGWs radiating away from the slope and perhaps overturning (breaking) and mixing locally if steep enough [52]. Another possibility is the generation of vertical vorticity $\zeta =\hat{\text{z}}\cdot \nabla \times \text{u}$ in the BBL underneath an along-slope current, as sketched and explained in figure 7. The vertical component of vorticity *ζ* is dynamically the most important one in QG theory, and at finite-*Ro* *ζ* is a major contributor to Ertel potential vorticity,

5.8 $$q_{E}=(f\hat{\text{z}}+\nabla \times \text{u})\cdot \nabla b,$$

at finite *Ro* where it enters multiplied by the stratification ∂_*z*_*b*. The flow and slope configuration in figure 7 generates *ζ*<0, and with either *s* or *V*_0_ of opposite sign, *ζ*>0 is generated. Often slope boundary currents separate from the boundary and move into the interior (e.g. at headlands or on the lee side of an island or seamount), carrying along their strong *ζ* and *q*_E_ anomalies and the associated strong shear. Once this fluid escapes the geometric constraint of an adjacent, impenetrable boundary and moves into the stratified interior, then shear instabilities associated with the *ζ* and *q*_E_ gradients arise, and the wake breaks up into vortices and other turbulence. The anomaly width in the sketch is ℓ_b_=*h*_b_/*s*, with *s* the bottom slope; e.g. if *h*_b_=20 m and *s*=10^−2^, then ℓ_b_=2 km, i.e. submesoscale. Beyond the process in the sketch with its uniform interior *V*_0_, horizontal inhomogeneities in *V* will produce inhomogeneities in ***τ***_b_ in the BBL that will add to the near-boundary vertical vorticity generation through (5.4) and *w*∼−curl[***τ***_b_]/*fρ*_0_ by Ekman pumping.

In [53,54], this phenomenological sequence is demonstrated for the California Undercurrent that flows poleward along the continental slope and then separates at the southern headland of Monterey Bay, CA, USA. The upstream BBL generation of negative *ζ* along the slope is so effective that the separating wake often has the strong signals of *ζ*<−*f* (when *f*>0) and *fq*_E_<0, and the latter relation satisfies the sufficient condition for centrifugal instability (CI; [55]),^9^ which is very efficient at lateral and diapycnal mixing. Thus, there is an outbreak of submesoscale activity in the separated current. This causes a dilution of the core *ζ* and *q*_E_ anomalies to subcritical values by mixing, the formation of interior coherent vortices, and subsequent flow organization into another type of long-lived SCV, Cuddies (California Undercurrent eddies). This sequence illustrates the BBL and boundary current path to SCV formation (e.g. as for Meddies; figure 3). Experience with realistic simulations shows many other examples of BBL vorticity generation, current separation and coherent vortex formation [9,58], where in anticyclonic *ζ* cases CI is a common intermediate stage [25]. The strong diapycnal mixing that arises during CI leads to an SCV core stratification that is nearly neutral. If through a sign reversal of the boundary current this process can in principle generate as many cyclones as anticyclones through vortex roll-up, when averaged over global mean and mesoscale boundary current and slope directions, then why are most detected SCVs anticyclonic? The answer does not lie in the QG approximation, where the sign symmetry (*ζ*_*g*_, *x*, *y*, *z*)↔(−*ζ*_*g*_, *x*,−*y*, *z*) means that cyclones and anticyclones evolve equivalently. One possibility at finite-*Ro* is that cyclones are more susceptible to disruption by perturbations than are centrifugally stable anticyclones, but so far this has only been shown in highly idealized models [59]. The observed anticyclonic prevalence of SCVs is still an unresolved theoretical issue.

Another unresolved dynamical issue is whether the combination of a flow along a bottom slope and stratification can lead to a suppression of ***τ***_b_ in the BBL through an advective rearrangement of *b* by up- or down-slope Ekman transport, sometimes referred to as an ‘arrested’ or ‘slippery’ Ekman layer [60]. If so, then it could potentially vitiate the boundary vorticity generation process described above. This issue is discussed further in §6b.

**Figure 8.**
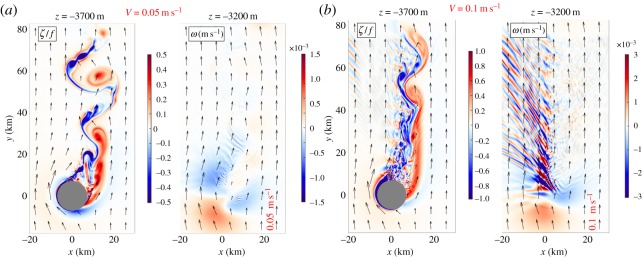
Snapshots of *ζ*(*x*,*y*)/*f* and *w*(*x*,*y*) at the indicated heights in simulated flow past a 600 m high seamount (the grey discs at *z*=−3700 m) above the sea-floor at *z*=−4000 m with a half-height diameter of 15 km. The solutions differ only in the speed of the incoming, horizontally uniform, barotropic flow, *V*_0_=0.05 (*a*) and 0.1 m s^−1^ (*b*). The former is firmly within a vortical wake regime, while the latter shows mixed vortical wake and lee IGW generation. Notice the doubled colour bar ranges with doubled *V*_0_ [61].

Finally, figure 8 shows *ζ* and *w* fields for a rotating, stratified flow *V*_0_ past a seamount. These two fields are good indicators, respectively, for the SMC vorticity and wake generation process and for the lee IGW generation process. Strikingly, with only a modest change in the speed of the incoming flow *V*_0_, the regime transitions from one with almost entirely an unstable vortical wake to another with significant IGW generation. The regime transitions for these phenomena have partly been mapped out [62], but as yet not enough attention has been given to the vortical regime as a generator of SMCs in general and SCVs in particular.

## 6. Other behaviours and effects

There are many other aspects of the ‘life cycle’ of SMC coherent structures and their associated finer scale turbulence and their effects on material *c* transport and mixing in the ocean [63]. Several are further discussed here.

### (a) Secondary instabilities

Echoing the admonition in §4b, a consideration of instabilities within the submesoscale range should start with QG, which has essentially two types: baroclinic instability of *V* (*z*) and horizontal (barotropic) instability of *V* (*x*), recognizing that often they can be mixed in *V* (*x*,*z*). These are both ‘inflection-point’ instabilities with a change of sign of ∂_*x*_*q*_*qg*_(*x*,*z*) within the domain as a necessary condition for instability (a.k.a. Rayleigh’s theorem). Their regimes of occurrence extend to finite *Ro*, albeit with some modifications expected. The former type is closely linked with the MLI generation of MLEs (§5a) that draws energy from the mesoscale potential energy in the SBL by $\bar{w^{\prime }b^{\prime }}>0$, and the latter is linked, at least by inference, to spirals on the sea (figure 2), drawing energy from the parent current by horizontal Reynolds stress work, $-\bar{u^{\prime }v^{\prime }}\partial_{x}\bar{v}>0$. Because both of these processes are rooted in momentum-balance dynamics, there is no deductive necessity for the instability products to exhibit imbalance and forward energy cascade, but neither is there a prohibition against it occurring at finite *Ro*.

A particular issue relevant to frontogenesis (§5b,c) is how frontal instabilities behave in the presence of a background strain field, i.e. in the midst of rapid frontogenesis. One paradigm is the suppression of barotropic instability in a *V* (*x*) flow [64]. Another paradigm with the opposite outcome is baroclinic instability of a buoyancy front in the surface layer, whose unstable growth rate *σ* increases faster with decreasing ℓ than does the frontal gradient ∂_*x*_*b* and thus is not suppressed by strain [65].

Other types of instability are inherently ageostrophic—absent in QG or even higher order BE models, but present in more general (hydrostatic) Primitive and Boussinesq equations models that retain the full ageostrophic acceleration, ∂_*t*_**u**_*a*_—and have the presumption of an imbalanced evolution even when the parent flow $\bar{\text{u}}$ is balanced. A perspective on this is provided by an analysis of the conditions for the non-integrability of a BE model, hence the necessity for a partly unbalanced further evolution [66], viz. the occurrence anywhere in the domain of ∂_*z*_*b*<0 (GI), *fq*_E_<0 (CI) or *f*(*f*+*ζ*−*S*)<0 (AAI). Here *S*^2^=(∂_*x*_*u*−∂_*y*_*v*)^2^+(∂_*y*_*u*+∂_*x*_*v*)^2^ is the square of the horizontal strain rate, with *fS*>0 by convention. All of these conditions are prohibited for small (*Ro*, *Fr*), and all can occur with *Ro*,*Fr*∼1. GI denotes gravitational instability (convection) associated with negative stratification, and AAI denotes anticyclonic ageostrophic instability, with reference to anticyclonic flow, *fζ*<0, as the location where this condition is most likely to occur. GI and CI are well-known processes, and both have finite-amplitude forward energy cascade and dissipation; they are particularly apt in the weakly (or negatively) stratified SBL [56,67]. AAI is a less widely familiar instability type but by now has a variety of adduced flow examples (see [68] and its references), including the ageostrophic instability mode in Eady’s flow [69,70]. Unlike GI and CI, AAI does not have a sharp onset condition in a general fluid model, but rather can exhibit an exponentially small unstable growth rate, $\sigma ∼|\nabla \bar{\text{u}}|\exp[-C/Ro\;]$ as Ro→0, which is also relevant to the asymptotic fuzziness of the slow (momentum-balanced) manifold (§4b; [10]), but with $\sigma ∼|\nabla \bar{\text{u}}|$ when *Ro*∼1, indicating that the fuzziness can become thick for SMCs outside this *Ro* limit.

The literature of ageostrophic instabilities, defined broadly for a fluid with finite *Ro* and *Fr*, is a very large one. Besides the types mentioned in the three preceding paragraphs, many examples exist for ‘resonances’ between neutral (*σ*=0) shear modes and IGW modes (including equatorial and coastal Kelvin wave modes) that coalesce when *Ro*∼1 through Doppler-shifting by *V* to yield an instability with *σ*>0 (e.g. [71]). Another important, well-known instability type is a Kelvin–Helmholtz (i.e. stratified shear) instability for *V* (*z*) with 0≤Ri≲0.25 (*Fr*>2) that likely plays a central role in the three-dimensional isotropization step in the forward energy cascade and thus is a reasonable demarcation of the small-scale limit of the submesoscale dynamical regime (figure 1). Even further into the regime of large *Ro* and *Fr* is a melange of other shear instabilities for $\bar{\text{u}(}\text{x})$ profiles, too numerous to map in a general way.

It is possible that these ageostrophic instabilities can provide a direct route from balanced mesoscale dynamics to unbalanced submesoscale behaviours. But present simulation experience suggests that it is far more common for the route to pass through the submesoscale coherent-structure generation processes in §5 and then, once the flow is already submesoscale with *Ro*, *Fr*∼1, for these secondary instabilities to arise, whether momentum-balanced or ageostrophic in type. As often true in fully developed turbulent flows, linear instability modes may not be readily recognizable. Instead they serve as dynamical demonstrations of the possibility for energy transfers from balanced into unbalanced currents, breaking the momentum-balance constraint *en route* to dissipation, although these proofs are mostly for steady, symmetric balanced flows, rather than for the more deformed evolving flows in turbulence.

### (b) Boundary potential vorticity injection or extraction

Potential vorticity *q* is a central quantity in GFD for rotating, stratified flows, because it is a composite dynamical variable that is advectively conserved; i.e.

6.1 $$\frac{Dq}{Dt}=n.c.t.,$$

where n.c.t. denotes non-conservative terms related to momentum and buoyancy diffusion and to boundary fluxes. *q* is a spatial differential functional of *b* and **u**; so its evolving spatial distribution provides an extra constraint on the relationship between velocity and buoyancy that is independent of any momentum-balance relation, e.g. (2.1). Furthermore, *q* figures prominently in some types of instability (§6a). In QG, a sign reversal of ∂_*x*_*q*_*qg*_ is a necessary condition for the inflection-point instabilities, and experience shows that they continue to finite-*Ro* even though Rayleigh theorems are less available. The condition *fq*_E_<0 is both necessary and sufficient for CI and sufficient for loss of BE integrability (§6d).

In QG, *q*_*qg*_ is a linear functional of *ϕ*, the advecting velocity is **u**_*g*_ and the n.c.t. are either externally specified or simple linear damping or diffusive flux divergences that convey the momentum and buoyancy boundary fluxes into the interior. For all these reasons, the interpretations of *q*_*qg*_ and its time evolution are relatively easy to understand.

In more general Boussinesq fluid dynamics, *q*_E_ in (5.8) is a nonlinear functional of *b* and **u**, and the n.c.t. are as well, viz.

6.2 $$\text{n.c.t.}=-\nabla \cdot [\nabla b\times F-(\;f\hat{z}+\nabla \times \text{u})B],$$

where F is the non-conservative force and B the non-conservative buoyancy source term, diffusive or otherwise [72]. Furthermore, because of the extra spatial derivatives in *q*_E_, the boundary fluxes for *q*_E_ are not fully determined from the boundary fluxes in *b* and **u** (which are sufficient for their well-posed fluid PDE problem). Thus, to evaluate how potential vorticity is influenced through these boundary fluxes, (6.1) must be integrated over a control volume, or else the boundary-normal component of the flux in (6.2) must be evaluated within the boundary-adjacent interior region. In either case, *q*_E_ fluxes can only be diagnosed *a posteriori*, either from (very difficult) measurements or from simulation model solutions. Because *b* is also advectively conserved, there is an integral constraint that, for a control volume between two isopcynal (constant-*b*) surfaces that might intersect the fluid boundary, the volume-integrated *q*_E_ can only change with time due to the normal n.c.t. fluxes through the intersected boundary area (sometimes referred to as isopycnal impenetrability [73]). Nevertheless, experience indicates that *q*_E_(**x**) is often strongly non-conservative through the effects of F and B in ways that can be subtle to interpret. Near the turbulent boundary layers or in the presence of a turbulent energy cascade to small scales, any coarse-graining (spatial smoothing) of *q*_E_ is unlikely to exhibit a conservative evolution.

Potential vorticity non-conservation has a strong connection to SMC dynamics, because of its close associations with the SBL and BBL where F and B are large (§5). Because *Ro* is often not small for SMCs, *q*_E_ in (5.8) and (6.1) and (6.2) are the relevant potential vorticity relations. Furthermore, where the wavenumber spectra are not very steep (§6d), the SMC imprint on *q*_E_(**x**) will be quite strong in relation to its mesoscale information.

In the SBL, the surface wind stress and buoyancy flux can cause large changes in the adjacent *q*_E_ when they occur where ∇_h_*b* and *ζ* are large at the surface, i.e. within SMC fronts, filaments and vortices. Surface cooling and heating act to extract and inject (i.e. decrease and increase) *fq*_E_ mainly by their effect on reducing or increasing the stratification ∂_*z*_*b*. Alternatively, wind stress with a down-front orientation (i.e. with $\hat{\text{z}}\cdot \tau_{s}\times \nabla_{h}b<0$) extracts *fq*_E_, and *vice versa* for an up-front stress [72]. The former situation can lead to *fq*_E_<0, and that can instigate additional turbulence through CI. This may even be compounded by the Ekman-current buoyancy flux (EBF) if it carries the near-surface dense water across the front to overlie lighter water (i.e. ∂_*z*_*b*<0) and instigate GI that reaches into the layer below. In this situation, the cross-front buoyancy flux divergence by the eastward Ekman transport in a vertically well-mixed surface layer is

6.3 $$\mathrm{EBF}=\frac{\tau_{s}}{f\rho_{0}}\times \hat{\text{z}}\cdot \nabla_{h}b=\frac{\tau_{s}^{y}}{f\rho_{0}}\partial_{x}b>0,$$

and it is approximately balanced by a vertical buoyancy flux divergence associated with a convective exchange with the layer below, with

6.4 $$\bar{w^{\prime }b^{\prime }}∼\mathrm{EBF}=\frac{u_{*}^{2}\partial_{x}b}{f}>0,$$

as in the simulations in [72,74] (cf. the other *wb* estimates in §5a–c). (Here the relations in red refer to the particular situation of a down-front wind.) This is more likely to happen if the Ekman layer is thin compared with the mixed layer within which ∂_*x*_*b* is fairly uniform with depth. When it does, if *fq*_E_<0 extends below the convective layer and CI remains active, then the sign of $\bar{w^{\prime }b^{\prime }}$ can reverse, and vertical shear production associated with the geostrophic shear can be significant, $-\bar{v^{\prime }w^{\prime }}\partial_{z}{\bar{v}}_{g}>0$ [75]. On the other hand, if the pycnocline beneath the SBL and SMC front is well stratified, then ∂_*z*_*b* can remain positive, and the effect of EBF is mainly to cause a bulk movement of the front rather than instigate convection, plus whatever other frontal instabilities may ensue.

A striking SMC event was observed at the Kuroshio–Oyashio confluence just east of Japan [8], where first a strain-induced frontogenesis created a very sharp surface front within the offshore jet, then a down-front wind instigated CI with an intense local cascade to dissipation *ε*, further leading to frontolysis. Other observed examples of *q*_E_ extraction and CI are at the sharp front that is the North Wall of the Gulf Stream (well away from its separation from the subtropical western boundary) when the surface stress is favourably aligned down-front [57,76].

In the BBL, the bottom stress ***τ***_b_ beneath an along-slope current *V*_0_ creates vertical vorticity *ζ* simply through the geometry of a turbulent shear layer over a slope (figure 7). (The bottom buoyancy flux is generally negligible, hydrothermal vents aside.) For *V*_0_ cyclonically rotated perpendicular to the up-slope direction (i.e. a counter-clockwise rotation in the Northern Hemisphere), the resulting *fζ* is negative (anticyclonic *ζ*), and *vice versa* for the reverse current direction (i.e. cyclonic *fζ*>0). Further, if ***τ***_b_ is horizontally variable, the vortex stretching process (5.4) further modifies *ζ* adjacent to the bottom. In computational simulations, this BBL *ζ* structure frequently leads to an effective SMC generation process when it is advected into the stratified interior by a boundary current separation followed by wake instability (§5d). Examples include the California Undercurrent [53,54] with *fζ*<0, CI, and SCV formation; the Gulf Stream in the Florida Strait, with a *fζ*>0 wake on the Florida side that forms a downstream cyclonic vortex street on the inshore side of the Gulf Stream [58] and a *fζ*<0 wake on the Bahamas side that induces CI with a large local dissipation rate *ε* in the interior [9]; and the northern slope of the Gulf of Mexico [77].

The sketch in figure 7 does not account for any dynamical influences from *b*(**x**), even though it does seem qualitatively apt as an explanation for the preceding SMC examples of wake instability. An open analysis question is how the large *ζ* value generated within the weakly stratified BBL transitions into a large *fq*_E_ anomaly during the boundary current separation process. Another open issue is the evolution of *b*(**x**) in a stratified BBL—with a substantial literature mostly for problems posed in an idealized two-dimensional [cross-slope *x*, *z*] configuration—that can reduce ***τ***_b_ and thus potentially suppress the BBL *ζ* and wake generation process, viz. an ‘arrested’ or ‘slippery’ Ekman layer over a sloping bottom in a stratified fluid (§5d). An along-slope interior flow *V*_0_ induces a cross-slope Ekman transport that is rotated in the cyclonic direction. In the presence of a $\bar{b}(z)>0$ stratification that intersects the sloping boundary, depending of the *V*_0_ direction, the Ekman transport implies either (i) a down-slope buoyancy advection that carries lighter fluid underneath denser interior fluid, induces convection that deepens the BBL thickness, and modifies *b*(*x*,*z*) in a way that has a geostrophic shear near the boundary that cancels the interior *V*_0_ at the boundary and reduces ***τ***_b_ or (ii) an up-slope buoyancy advection of dense water that strengthens the near-boundary stratification, shrinks the BBL thickness and reduces ***τ***_b_ [78]. At the present time, there is neither a satisfactory theoretical synthesis of these two competing processes, nor a full characterization in terms of *q*_E_ and its boundary injection.

### (c) Frontal arrest

Assuming the well-posedness of the Navier–Stokes equations, then the finite-time singularity predicted for two-dimensional frontogenesis in balanced models (§5b,c) will not occur. One possibility is a frontal arrest associated with molecular viscosity *ν*_m_ at a very small horizontal scale of, e.g. $\ell_{m}∼\sqrt{\nu_{m}/\alpha }$ for strain-induced frontogenesis, but more likely is some kind of frontal instability that provides opposing eddy fluxes that accomplish the arrest at a larger scale than ℓ_m_. Buoyancy diffusion with *κ*_m_ could have a similar effect. In a computational model with a necessary but usually ad hoc eddy viscosity *ν*_e_, frontogenesis can drive a front down to near the grid scale, where an analogous viscous arrest occurs at ℓ_e_≫ℓ_m_, but this is not a convincingly physical depiction of the arrest process.

As frontogenesis proceeds, frontal gradients grow, and shear instabilities become ever more explosive with growth rates *σ*∼|∇**u**|. What kind of frontal instability can accomplish an arrest? There are multiple candidates. In a low-mode Galerkin projection model of frontogenesis and baroclinic frontal instability, horizontal eddy buoyancy flux $\bar{u^{\prime }b^{\prime }}$ provides an arrest in $\partial_{x}\bar{b}$ [79], albeit in less than fully three-dimensional fluid dynamics. This suggests that MLI and MLEs might sometimes suffice, although as yet this has not been demonstrated. In an observed Kuroshio frontogenesis event (§6b), frontal arrest and even relaxation occurred after the onset of CI followed by a strong cascade to dissipation [8]. In a simulation of dense surface filaments in the Gulf Stream, a life cycle sequence is demonstrated: frontogenesis; horizontal shear instability and eddy momentum flux $\bar{u^{\prime }v^{\prime }}$; frontal fragmentation; and finally ∇*b* relaxation and decay. Because the frontal arrest occurs near the model’s horizontal grid scale, the influence of *ν*_e_ cannot be excluded [36]. Another possibility is that SBL turbulence forced by surface wind stress and buoyancy flux could provide horizontal stirring and mixing down the frontal gradients in **u** and *b* as if the latter were passively advected, but the assumption of passivity during frontogenesis is dubious.

For the most part, the processes of SMC frontal arrest are still to be discovered. If the arrest happens on small SMC scales with $\ell ≳h_{b}$, then an LES model is necessary to simulate the three-dimensional, non-hydrostatic turbulence involved in the arrest (as well as in the SBL itself). This implies a quite large calculation to span the range from metres to kilometres, and the SMC component must be either highly idealized or controlled by a long sequence of nested grids.

An early example is the phenomenon of TTW-induced frontogenesis of an idealized dense surface filament, where the design of the three-dimensional LES configuration [80] is guided by a larger scale, two-dimensional simulation using a parametrization model for *ν*_v_,*κ*_v_(*x*,*z*) [37]. The initial width of the dense surface filament 〈 *b*〉(*x*,*z*) (where brackets denote an average in the along-filament direction *y*) is about 4 km in a weakly stratified surface layer 60 m thick above a stratified pycnocline, i.e. qualitatively similar to the sketch in figure 5. The maximum speed of the along-front jets in 〈 *v*〉 is ≈0.25 m s^−1^. A uniform wind stress $\tau_{s}=\rho_{0}u_{*}^{2}$ is present, which drives a turbulent Ekman layer in the far-field well away from the filament; for the present example, the wind is to the north, so the near-surface Ekman currents are to the northeast. The problem is initialized first with a spin-up to a *x*-uniform turbulent Ekman layer. Next a nudging to an idealized *T*(*x*,*z*) filament structure is added for a couple of hours while a geostrophic adjustment occurs. Then the flow is released for a free evolution. The evolution is frontogenetic, as expected for the TTW secondary circulation with a surface convergence in 〈 *u*〉(*x*,*z*) and central downwelling in 〈 *w*〉(*x*,*z*) (§5c). When the frontal width reaches a cross-filament scale ℓ of a few hundred metres, a horizontal shear instability of the along-filament flow 〈 *v*〉(*x*,*z*) emerges from the SBL turbulence, and its cross-filament eddy momentum flux begins to induce a frontal arrest. The *y*-averaged northward momentum balance is, partially,

6.5 $$\partial_{t}\langle \;v\rangle =-\partial_{x}[\langle \;u\rangle \;\langle \;v\rangle +\langle \;u^{\prime }v^{\prime }\rangle ]+\cdots ,$$

where the prime denotes a deviation from the *y*-average. The first right-side term is the divergence of the frontogenetic momentum flux by the Ekman currents and secondary circulation, and the second term is the frontolytic turbulent eddy momentum flux. The along-front wavenumber spectrum of 〈 *u*′*v*′〉 is dominated by scales ≫*h*_b_; i.e. it is more like a submesoscale secondary instability than boundary-layer turbulence.

**Figure 9.**
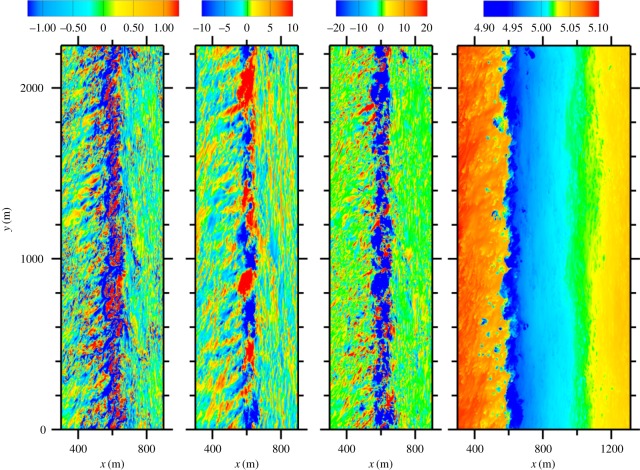
TTW-induced frontogenesis of a dense SBL filament at a time of incipient frontal arrest (i.e. *t*=4.2 h after the completion of the initialization phase) with a northward (down-front) wind stress in an LES simulation: (left to right) (*x*,*y*) snapshots of *w*/u_*_, *v*^′^/u_*_, $u^{\prime }v^{\prime }/u_{*}^{2}$, and *T* [C] near the surface. The colour bars in the first three panels are saturated with some extrema outside the listed ranges [80].

**Figure 10.**
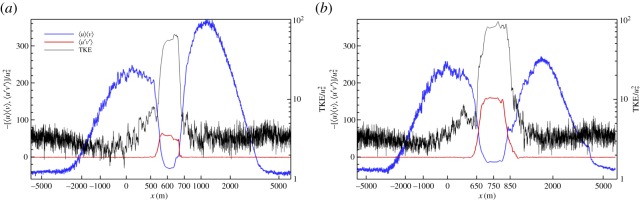
Normalized momentum fluxes, 〈*u*〉 〈*v*〉 and 〈*u*′*v*′〉 in (6.5), and turbulent kinetic energy (TKE) near the surface during the frontal arrest phase for TTW-induced frontogenesis of a dense SBL filament: (*a*) *t*=4.2 h after the end of the initialization (i.e. the same time as in figure 9) and (*b*) *t*=6.7 h. The abscissa is a stretched *x*-coordinate to show both the near and far fields of the filament centred at the place of maximum ∂_*x*_〈 *v*〉. The jitter in the curves is sampling error associated with the finite domain in *y* [80].

Figure 9 shows several near-surface fields at a time when the arrest is starting, about 4 h after the initialization phase is completed. With *b*∝*T*, the cold filament width has now shrunk to about 500 m, and it has moved eastward by Ekman current advection from its initial centre at *x*=0, but the strongest activity is in a narrower ≈200 m strip on its west side; this *x*-asymmetry is due to the eastward Ekman current augmenting the otherwise odd-symmetric TTW 〈 *u*〉 in the advection of 〈 *T*〉. The SBL turbulence with ℓ∼*h*_b_ is most intense around this west edge of the filament, and the weaker patterns on either side have a different character at least until far away from the filament. In *v*′ and *w*, a larger scale in *y* is also evident, and the negative product *u*′*v*′ indicates its prevalent contribution to the frontolytic eddy momentum flux 〈 *u*′*v*′〉<0. Further into the integration period, the surface *T* anomaly begins to weaken, then ∂_*x*_〈 *v*〉 relaxes and meanders in *y* develop along the filament axis. This later relaxation and decay phase is slower than the frontogenesis and arrest phase, lasting more than a day. Early in the integration 〈 *u*〉 〈 *v*〉 is much stronger than 〈 *u*′*v*′〉 in (6.5), implying net frontogenesis. During the arrest phase, these two fluxes have comparable, opposing gradients where the eddy flux divergence grows to match and then exceed that of the secondary circulation (figure 10). During the prolonged relaxation phase, both of the momentum fluxes and the turbulent kinetic energy (TKE) decline in a somewhat disorderly way. In addition to the eddies with a longer *y*-scale, the smaller scale turbulence is also energized in the western filament edge zone, with the TKE a factor of 20–30 higher than in the far field.

The LES study [80] includes several examples with different wind directions and the presence or the absence of surface gravity waves in wind-wave equilibrium (WWE; §6g). In all cases, the TTW-induced frontogenesis and frontal arrest occur at a small submesoscale ℓ that is a few hundred metres, but the particular turbulent flow structures involved are noticeably different, indicating that a broad range of arrest behaviours may be expected. Furthermore, the example here is for a down-front wind, where the onset of CI due to the extraction of *q*_E_ by the wind and the occurrence of convection induced by the eastward Ekman buoyancy flux (EBF; §6b and [74]) are not the primary behaviours. At this point, it is only speculation about why not, but the rate of frontogenesis ∂_*x*_*u*, the strength of the pycnocline stratification *N*, and the ratio of the filament *v*_*g*_ and Ekman velocities, $∼h_{b}^{2}\partial_{x}b/u_{*}^{2}$—all with rather high magnitudes here—seem likely to be relevant.

### (d) Breakdown of balance and forward energy cascade

The concept of an inertial cascade range is central to turbulence theory. It is defined as a stationary, scale-invariant flux of some property by the advective operator (e.g. for kinetic energy, the spectrum flux *Π*_KE_(*k*) is constant over some extensive range in wavenumber *k*). This in turn specifies a consistent power-law spectrum shape, e.g. *E*_KE_(*k*). These ranges are related to the inviscid integral conservation of a quadratic property (e.g. **u**^2^) whose variance can be advectively exchanged between scales. Rather few inertial cascade ranges are known: (a) in three-dimensional isotropic homogeneous turbulence (a.k.a., Kolmogorov’s regime), kinetic energy cascades to small scales with a constant flux *Π*_KE_ equal to the viscous dissipation rate *ε*>0, hence *E*_KE_(*k*)∼*ε*^2/3^ *k*^−5/3^; (b) in two-dimensional isotropic homogeneous turbulence, kinetic energy (inverse) cascades to larger scales at a rate $\Pi_{\mathrm{KE}}(k)=-\hat{\varepsilon }<0$, hence $E_{\mathrm{KE}}(k)∼{\hat{\varepsilon }}^{2/3}\;k^{-5/3}$, or enstrophy ${\bar{\zeta }}^{2}$ cascades to small scales at a rate *Π*_ens_=*η*>0, hence *E*_KE_(*k*)∼*η*^2/3^*k*^−3^; (c) in geostrophic (i.e. for the QG model), horizontally isotropic, homogeneous turbulence, the cascade ranges are analogous to those in two-dimensional except for total energy (kinetic plus available potential) and for potential enstrophy $\bar{q_{qg}^{2}}$ with a three-dimensional wavenumber in which *k*^*z*^ is rescaled by *f*/*N* [4]^10^ and (d) in horizontally homogeneous and isotropic SQG turbulence, there are again two inertial cascade ranges, an inverse one for volume-integrated total energy and a forward one for surface buoyancy variance, $\bar{b{\left(x,y,0\right)}^{2}}$ [32], with the latter potentially more relevant for SMCs, because it is accompanied by the formation of small-scale surface frontal features. Remarkably, in SQG the surface buoyancy variance and surface horizontal kinetic energy $\bar{{\text{u}}_{h}{\left(x,y,0\right)}^{2}}$ spectra have a constant ratio for every horizontal wavenumber *k*_h_, so the forward buoyancy spectrum flux *Π* is equivalent to an inverse velocity spectrum flux −*Π* with *Π*>0, and both quantities have a spectrum shape ∼*k*^−5/3^_h_; this regime has vanishing **u** and *b* variance dissipations as Re→∞ [82]. Thus, with the exception of (a) that is inapplicable to rotating stratified flows, none of the other inertial cascade ranges (b)–(d) represents a route to dissipation, hence they not an apt model for the submesoscale energy flow in figure 1. Perhaps not coincidentally, (b)–(d) are also forms of momentum-balanced dynamics with **u** a spatial-differential functional of *ϕ* (cf. (2.1) for QG).

A variety of differently formulated simulations demonstrate that there is a relevant forward energy cascade range for SMCs that probably should not yet be called an inertial range, pending further clarification about its asymptotic behaviour in Ro,Fr→0,Re→∞. It arises in two seemingly distinct situations.

The first situation is the turbulent SBL [38,83]: both $\bar{b^{2}}$ and $KE_{h}=\frac{1}{2}\;\bar{{\text{u}}_{h}^{2}}$ in the SBL have horizontal spectrum shapes close to ∼*k*^−2^_h_ (i.e. slightly steeper than the SQG inertial range). Neither of these quantities is a conservative invariant because of exchanges with the underlying interior. The buoyancy variance has a forward cascade, *Π*_b_(*k*_h_)>0, for all *k*_h_ up to the high-wavenumber dissipation range. For surface kinetic energy, *Π*_*KE*_h__(*k*_h_) changes sign within the nearly inviscid *k*_h_ range; i.e. <0 (inverse) for the smaller *k*_h_, and >0 (forward) for larger *k*_h_; N.B. the latter behaviour is unlike the SQG inertial range. The cross-spectrum ${\bar{w^{\prime }b^{\prime }}}_{k}$ is >0 (restratification flux and kinetic energy generation) for smaller *k*_h_, and it is <0 (kinetic energy depletion) for larger *k*_h_. Note that this large-*k*_h_ ${\bar{w^{\prime }b^{\prime }}}_{k}<0$ behaviour is different from the MLI and frontogenesis processes in §5, and it occurs on smaller scales as part of the ensuing forward energy cascade. The *Π*_KE_h__(*k*_h_)>0 result is inherently ageostrophic in the sense that it depends on calculating the flux with the total **u** in the flow not just the **u**_*g*_ component.

The second situation is more relevant to the oceanic (or even atmospheric) interior, and it has mostly been simulated in idealized flows with horizontal periodicity and spatial homogeneity and without either SBL or BBL turbulence. It is a dual forward cascade in volume-integrated kinetic and available potential energies. Neither of these is a conservative integral invariant, although their sum, the total energy, is, and available potential energy is not a quadratic functional of *b* for non-uniform stratification *N*(*z*). Nevertheless, with three-dimensional periodicity and homogeneity, both for flows decaying from balanced initial conditions or in a statistical equilibrium with random forcing at a small wavenumber [84–87] or even with steady mean wind stress [88], the smaller *k*_h_ range has a mostly balanced flow (i.e. |**u**_*g*_|>|**u**_*a*_|) with a steep spectrum shape ∼*k*^−3^_h_ and with *Π*_KE_<0 (inverse cascade). At larger *k*_h_, the spectrum shapes become shallower, close to *k*^−5/3^_h_, with nearly constant values for *Π*(*k*_h_)>0, and with ${\bar{w^{\prime }b^{\prime }}}_{k}<0$ (kinetic energy depletion); this feature in *E*(*k*_h_) is sometimes referred to as the ‘nose’ of the spectrum because it protrudes from an intermediate *k*_h_ out to where it steepens in the dissipation range. In this higher-*k*_h_ cascade range, *Ro*_*k*_ is not small, and it increases with *k*_h_; so the flow is not highly momentum-balanced. On the other hand, the identified IGW activity in this spectrum range is quite weak [85]. In the magnitudes of both, the spectrum *E* and the spectral flux *Π* in the higher-*k*_h_ range, the kinetic energy component is larger than the available potential energy component. The transition *k*_h_ value between these two regimes shrinks with an increasing spectrum-peak value of *Ro*, and the magnitude of the *Π*>0 value increases with *Ro*. In an equilibrium turbulent Eady flow with solid top and bottom boundaries, the same behaviours are found [89,90]. Furthermore, even in statistical-equilibrium, stratified turbulence without rotation but with small horizontal Froude number *Fr*_h_=*V*/*N*ℓ≪1, the same dual forward-cascade behaviour occurs [91], indicating that there is a seamless transition between intermediate scales with moderate *Ro*_*k*_ with important rotational effects and smaller ones with *Ro*_*k*_ large and small rotational influences.

These surface and interior dual cascade regimes are more similar than different in their behaviours. All of their properties are, for now, experimentally determined with some quantitative uncertainties (e.g. in the spectrum exponent) and without a cogent theoretical explanation. The kinetic and potential energy cascades are not independent because ${\bar{w^{\prime }b^{\prime }}}_{k}\neq 0$. It cannot be ruled out that there are weak *k*_h_ trends in *E*(*k*_h_), *Π*(*k*_h_), and ${\bar{w^{\prime }b^{\prime }}}_{k}$, with perhaps smaller or zero trends in the total energy spectrum shape and spectrum flux, bringing the combined total energy closer to having a true inertial cascade range; i.e. this behaviour may or may not be truly asymptotic in some double limit of Ro, Fr→0 and Re→∞. The relatively shallow spectrum slopes in *E*(*k*_h_) are indicative of a breakdown of momentum-balance, for which there is no general explanation, although the ageostrophic instability processes (§6a) provide a partial explanation. These cascades are anisotropic with *h*≪ℓ when *f*≪*N*, and, if not limited by the diffusion due to a limited *Re* value in the simulations, their ranges terminate at large *k*_h_ for ℓ∼*V*/*N*∼*h*, i.e. where Kelvin–Helmholtz instability and isotropic overturning motions occur. For even larger *k* values, the forward cascade to dissipation will occur, and Kolmogorov’s paradigm of the three-dimensional isotropic, homogeneous inertial cascade (with $Ro_{k},\;Fr_{k}\to \infty$) seems apt.

These SBL horizontal spectrum shapes are fairly well confirmed in the ocean [38], but the interior ones are not, partly because such interior SMC measurements are rare and easily confused with IGW phenomena, and partly because most realistic simulation models still show steeper spectrum shapes. The idealized turbulence simulations demonstrate the theoretical viability of an interior forward energy cascade, but it is still unknown when and where they should be manifest in the ocean, i.e. apart from boundary-generated SCVs, it is not yet known how active SMCs are in the oceanic interior.

A final caution should be stated: surface waves and IGWs undergo their own turbulent cascades (i.e. wave turbulence; [92]), and surprises may arise when this occurs simultaneously with SMC turbulence (§6f).

### (e) Seasonal and geographical variability

Mesoscale eddies show significant geographical variation in their size and amplitude but little seasonal modulation in most locations, as has been well measured at the surface by geostrophic inference from satellite altimetry [93]. There is no comparable global measurement system for SMCs. Simulations must be done regionally to have a fine enough grid resolution for SMCs to emerge, and as yet the examples are piecemeal.

Because SMCs are created from mesoscale eddies, they no doubt show some degree of correlated geographical variability. A more specific guide to SBL SMC activity comes from the scaling formulae (5.2), (5.3), (5.5) and (5.6): greater activity is predicted for bigger surface buoyancy gradient ∇_h_*b*, SBL depth *h*_b_, ambient strain rate *α* and SBL eddy viscosity *ν*_v_. These imply covariances with both stronger mesoscale eddies and with stronger boundary-layer mixing, typically associated with winter weather. The winter peak of SMC activity is confirmed in simulations [94–96]. There is also the prediction of weaker SMC activity with higher latitude (larger *f*), other factors being equal, but this effect has not been well surveyed. The equatorial ocean is likely to be atypical because of the breakdown of even geostrophic balance, and little is known about mid-oceanic SMC activity there. Even in summer with small *h*_b_ due to surface heating, SMC activity persists at some level albeit with weaker *V* and smaller ℓ [50]; the latter has the effect of pushing the emergence of SMCs below the grid-resolution scale for most simulations to date.

Overall, these are still early days for learning about SMC geographical variability in both measurements and simulations. Major river inflows provide a different source of ∇_h_*b*, hence can increase the SMC activity, and their seasonal peaks do not always match those of winter cooling [97]. Strong currents and Rings are special sites for some types of SMCs, though they too have seasonal variability associated with *h*_b_ [36]. Over shallow topography near coasts and islands, the wake-instability generation process will also be an important SMC source near the surface (e.g. in the tropical Western Pacific archipelagos). At depth, this process should generate SMCs wherever the combination of bottom mesoscale currents and topographic slopes occurs, followed by dispatching the locally generated SCVs for wider dispersal. Finally, the high-latitude regions have generally weak stratification and a small baroclinic deformation radius ℓ_d_<10 km, which are likely to give rise to distinctive SMC behaviours.

### (f) Inertia-gravity wave coupling

Perhaps the hoariest uncracked nut is the question of SMC–IGW coupling. Experience shows a modest increase in IGWs—in so far as they are recognizable from approximate conformity with their dispersion relation—with increased grid resolution and SMC energy level in realistic simulations posed without high-frequency wind forcing or tides (the principal IGW generation processes), but the IGWs remain weak compared with the SMCs. This is consistent with the analytical conclusion that spontaneous IGW emission by momentum-balanced flows is not a strong process for small *Ro*, and even for *Ro*∼1 but *Fr*≪1, it is weak [10].^11^ Nevertheless, a variety of idealized simulations have exhibited some degree of spontaneous emission, especially where the momentum-balanced flow component is rapidly evolving, e.g. in frontogenesis [98]. However, the small-scale wave activity is quite weak in randomly forced, homogeneous turbulence with a moderate *Ro* value (§6d; [85]).

In the ocean, there is abundant IGW activity due to tidal and transient wind forcing. The interaction of mesoscale eddies with IGWs is fairly well understood: strong eddies can refract IGWs [99] and provide catalytic interactions that enhance the IGW forward energy cascade [81] (also related to ‘wave capture’ [100]), and lee IGWs can be generated by eddy currents over topography [52] (e.g. figure 8). For SMCs, there are some examples of high-strain IGW emission from fronts [101–103] and of high-frequency forcing exciting inertial currents and stimulating the SMC forward energy cascade rate and *ε* [104], but these stories are far from complete. It seems likely that SMC–IGW coupling often is manifested in small-scale currents that are neither highly momentum-balanced nor clearly propagating, i.e. showing a hybrid behaviour unlike the simpler forms of either alone.

### (g) Surface wave effects

Surface gravity waves span the oceans, and their orbital velocities are dominant in at least the upper part of the SBL. They are generated primarily by surface winds, and an important reference concept is wind-wave equilibrium (WWE), where the rates of wind energy and stress transfers into the wave field are matched by the dissipation and loss of wave momentum into oceanic currents mainly through wave breaking. Because the peak wavenumber of the wave energy spectrum in WWE is associated with a small slope in the sea surface (implying an approximate linear propagation dynamics) and because the peak wave period is much shorter than a current evolution time, a rational asymptotic theory of wave-averaged dynamics can be derived for the currents [105,106]. In addition to the usual fluid dynamics, wave-averaging yields additional Stokes–Coriolis and Stokes vortex force terms in the momentum equation, $-(\hat{\text{z}}f+\nabla \times \text{u}){\text{u}}_{s}$, and a Stokes advection term in the buoyancy and material concentration equations, −**u**_s_⋅∇*c*, where **u**_s_ is the Stokes drift that is the Lagrangian mean velocity for surface waves. In WWE, a turbulent Langmuir number, $La=\sqrt{u_{*}/V_{s}}$ (with $u_{*}=\sqrt{\tau_{s}/\rho_{0}}$ and *V*_s_ the magnitude of Stokes drift at the surface), has a value around 0.3, indicating that these wave-added terms are significant in relation to the wind-driven (Ekman) currents in the SBL.

Surface wave effects on currents are well known to be important in SBL turbulence, in the regime called Langmuir turbulence [107]; they are also important in the littoral zone where shoaling waves break and accelerate along-shore and rip currents [108]. A much newer proposition is that they are also important for SMC dynamics in the SBL.

Scaling analysis of the wave-added terms indicates that the Stokes vortex force provides a correction to geostrophic balance with a relative amplitude^12^

6.6 $$\epsilon =\frac{h_{b}}{h_{s}}\;La^{-2}Ro$$

[109,110]. The first factor is the ratio of the SBL depth *h*_b_ to the vertical decay scale *h*_*S*_ for the **u**_s_(*z*) profile, which is typically less than 10 m; i.e. this factor is large. The second factor is also large in WWE (and even larger in ‘old’ seas with *La*<0.3). The third factor *Ro* represents the usual effect of advection as a correction to geostrophic balance, which is small for mesoscale eddies and often around 1 for SMCs.

A steady conservative solution with the wave-averaged forces and buoyancy advection occurs for a two-dimensional front or filament in *b*(*x*,*z*) and a uniform, steady Stokes drift **u**_s_(*z*). It has *u*=−*u*_s_, *w*=0, and the following diagnostic *x*- and *z*-momentum balances:

6.7 $$-f(v+v_{s})=-\partial_{x}\pi +v_{s}\partial_{x}v\;\mathrm{and}\;\partial_{z}\pi =b+v_{s}\partial_{z}v,$$

where *π* is a generalized pressure normalized by *ρ*_0_ that contains wave and current Bernoulli terms [109]. It is an obvious generalization of the geostrophic, hydrostatic balance in (2.1) due to the presence of surface waves with *v*_s_≠0. As in both (2.1) and (5.6), it is an under-determined system: given *b* and **u**_s_, it is diagnostic for **u** and *π*; or, as solved for in [109], given an initial *q*_E_(*x*,*z*) in the absence of surface waves, how do *b*, *π* and **u** conservatively adjust to a new steady state with their arrival?

Thus, surface wave effects matter for surface SMC dynamics. As yet few relevant solutions have been obtained for the wave-averaged equations. For an otherwise geostrophically balanced, steady, conservative front or filament, the wave effects break the primary cross-axis symmetry in *b* and *v*_*g*_ (e.g. odd in *x* for *b* and even for *v*_*g*_ in an idealized front and *vice versa* in a filament) by spinning up opposite-symmetry components during a conservative adjustment to arriving surface waves. Thus, the Stokes forces modify the shapes of steady, momentum-balanced fronts and filaments [109]. The linear eigenmodes for baroclinic and centrifugal (CI) instability in the SBL are altered modestly by the Stokes forces, as is their subsequent finite-amplitude evolution [111]. In frontogenesis events, both for a field of MLEs [112] or for TTW-induced dense filament sharpening [80], the Stokes forces significantly influence the evolving flow patterns. Obviously, much more exploration of wave effects on SMCs is needed.

### (h) Lateral dispersion

A nearly universal behaviour in turbulent flows is the spatial spreading among neighbouring fluid parcels on average, which is a Lagrangian behaviour. This implies a stirring of the associated material concentration *c* with the surrounding environment. A common measure of this effect is the relative dispersion

6.8 $$D^{2}{\left(t)=\langle \;(\text{x}(t)-{\text{x}}^{\prime }(t)\right)}^{2}\;\rangle ,$$

where the average is over labelled parcel pairs (**x**,**x**^′^) released at *t*=*t*_0_ in some small, finite region. The spreading is indicated by the monotonic growth of *D*^2^(*t*) in an ensemble of releases. A random walk is a paradigm for dispersive behaviour, with *D*^2^∼*t*, and this functional dependency is also characteristic of material diffusion.

There are some partial exceptions to this expectation for SMC currents. For example, parcel releases inside an SCV (figure 3) that does not leak its interior parcels will have a bounded *D*^2^. Another example is a patch of buoyant surface parcels released in an SMC flow with strong surface convergence lines (figures 2 and 6). The parcels will gather into a much smaller area occupied by the width of the local convergence lines, at least for as long as these coherent flow structures persist. Even in this situation, *D*^2^ might grow due to increasing distance among separate structures or due to parcel spreading along the lines. Nevertheless, for general releases and long times, increasing *D*^2^(*t*) is still expected, recognizing that such a bulk measure masks some interesting transient, local patterns.

In a power-law wavenumber spectrum regime with *E*_KE_(*k*)∼*k*^−*β*^, there are associated behaviours for *D*^2^(*t*), as well as for the Lagrangian diffusivity, $\kappa_{L}=\frac{1}{2}\partial_{t}D^{2}$, and the finite-scale Lyapunov exponent (FSLE) *λ*, which expresses the exponential rate that parcel pairs increase their separation distance by a specified multiplicative factor [113]. These quantities can alternatively be expressed as functions either of *t* or of the growing patch size $d(t)=\sqrt{D^{2}}$. For *β*≥3, relevant to the geostrophic potential-enstrophy inertial cascade range (§6d) and decaying geostrophic turbulence, *D*^2^(*t*) shows an exponential growth and *κ*_*L*_(*d*) and *λ*(*d*) are independent of *d*. The interpretation is that the largest eddies at the low-*k* end of the spectrum range dominate the spreading behaviour throughout the range; i.e. the dispersion dynamics at *d*(*t*) is non-local in *d*. SMCs have shallower spectra, with estimated *β* values of $\frac{5}{3}$ to 2 (§6d). This implies the following power-law dependencies:

6.9 $$D^{2}(t)∼t^{4/\left(3-\beta \right)},\;\kappa_{L}∼t^{\left(1+\beta \right)/\left(3-\beta \right)}\;\mathrm{or}\;d^{\left(1+\beta \right)/2},\;\lambda (d)∼d^{(\beta -3)/2}.$$

For $\beta =\frac{5}{3}$, which is also the scaling in Kolmogorov’s isotropic regime, these four exponents are, respectively, $\left(3,\frac{3}{2},\frac{4}{3},-\frac{2}{3}\right)$. This regime is called Richardson’s regime, with its famous $\kappa_{L}(d)∼d^{\frac{4}{3}}$ law. Here the dispersion is scale-dependent and local in *d* in the sense that local velocity differences *V*_d_ control the dispersion behaviour. For *β*=2, the four exponents are $\left(4,3,\frac{3}{2},-\frac{1}{2}\right)$.

In an experiment that deployed an unprecedentedly large number of surface drifters in the northern Gulf of Mexico [114], the diffusivity and FSLE estimates are fairly well fit by the power-laws *κ*_L_(*d*)∼*d*^4/3^ and *λ*(*d*)∼*d*^−2/3^ over a scale range from hundreds of metres to tens of kilometres, i.e. for the SMCs. Owing to estimation error it may be difficult to distinguish these exponents from $\frac{3}{2}$ and $-\frac{1}{2}$, respectively. Nevertheless, It is unambiguous that SMCs add a scale-local dispersion increment above the contribution from mesoscale eddies, and there is a general consistency between the simulated shallow spectrum shapes and the measured dispersion behaviour in this SMC SBL soup regime.

## 7. Summary and prospects

SMCs spontaneously emerge from mesoscale eddies and boundary currents, especially in the vicinity of the SBL and BBL. They are generated through instabilities, frontogenesis and topographic wakes. They are partly constrained by geostrophic, hydrostatic momentum-balance, but also break this balance at smaller scales and exhibit a forward energy cascade to dissipation and diapycnal mixing. Neither of the dissipation and mixing rates is yet accurately quantified for SMCs on a global scale, although local examples show they can be significant.

The science of SMCs is still quite young compared with other GFD subfields, e.g. general circulation, mesoscale eddies, weather prediction, boundary layer turbulence, IGWs and surface waves. So far little has been measured for SMCs because of the sampling difficulties discussed in §1. Computational simulations have done much to demonstrate SMC phenomena, but there remains a gap in simulating the smaller end of their scale range (i.e. for ℓ≈50–500 m), in part because the border between hydrostatic and non-hydrostatic SMC dynamics lies within this range. Some recent pioneering studies [80,115,116] extend the domain size in LES of SBL turbulence with idealized SMC flows, but the computational technology for nesting realistic simulations down to this scale of non-hydrostatic dynamics is not yet well developed.

Finally, from the various remarks above, I hope it is apparent that the theoretical explanations for many SMC phenomena are far from complete. An incomplete list of compelling unresolved issues is the following:

— approximate slow-manifold behaviour of momentum-balanced flows and how it breaks down;
— rates of forward energy cascade and diapycnal mixing in the SMC dual cascades;
— maintenance of surface biological productivity by SMCs;
— SMCs in strong currents compared with the more prevalent SBL soup regime;
— momentum-balance, forward energy cascade and SMC dynamics in general near the equator where f→0;
— ageostrophic advective dynamics in SMC secondary circulations;
— SMC turbulence in the oceanic interior;
— SMC frontal instabilities that lead to fragmentation and/or arrest;
— partition between vorticity generation and drag suppression by bottom stress ***τ***_b_ in the BBL;
— partition between SMC vortical and IGW lee-wave wakes in topographic flows;
— coupling between IGWs and SMCs;
— anticyclonic prevalence among observed SCVs;
— coupling between surface gravity waves and SMCs in the SBL;
— reconciling surface convergence with ensemble horizontal dispersion in a turbulent SMC soup; and
— long-range lateral transport by SCVs in the oceanic interior.

## Footnotes

1 Of course, energy is injected at the surface at all scales. For the most part, this leads to local surface-wave, boundary-layer, IGW, and perhaps even SMC generation that have more direct routes to dissipation, being much less influenced by the momentum-balance constraints discussed in this section. Some aspects of submesoscale injection are discussed in §6b.

2 Notice that these diagnostic momentum-balances (i.e. without any ∂_*t*_) under-determine the fields. The complete dynamical system, called Quasigeostrophy, has additional evolution and mass-balance equations.

3 This scaling estimate assumes a constant ‘mixing efficiency’, as has commonly been done in interpreting oceanic microstructure measurements [11]. It is an open question whether this assumption is valid more widely, e.g. for an SMC forward energy cascade.

4 The SQG model is relevant only for ∇_h_*b* at the approximately flat upper surface. The bottom is geometrically irregular and often has a much weaker |∇_h_*b*|.

5 Regularity of the Navier–Stokes equations is one of the Clay Millennial unsolved problems in mathematics; http://www.claymath.org/millennium-problems.

6 This estimate is obtained from the QG ‘omega equation’, where the relevant terms in a two-dimensional configuration are $[\;f^{2}\partial_{z}^{2}+N^{2}(z)\partial_{x}^{2}]w=\alpha f\;\partial_{z}\partial_{x}v_{g},$ and an assumption that *N*_s_*h*_b_≤*f*ℓ.

7 In [42], the terminology of turbulent thermal wind is used for a different situation, viz., one that is horizontally homogeneous in the presence of a uniform $\partial_{x}\bar{b}\neq 0$. It is more relevant to a MLE turbulent equilibrium (§5a) than it is to a local submesoscale coherent structure.

8 For *Ro*∼1, due to ageostrophic momentum and buoyancy advections, the secondary circulations in fronts and dense filaments generally have larger amplitudes than in the linear estimators in (5.5) and (5.7) [49].

9 CI here is used here as a generic term for instabilities and turbulence enabled by *fq*_E_<0 and ∂_*z*_*b*≥0, rather than distinguishing among its variants associated with different contributing terms in *q*_E_. Some authors distinguish symmetric instability [56,57], which does not require *ζ*<−*f*, and neither is it restricted to symmetric flows and instability modes (i.e. with ∂_*y*_=0).

10 In [81], this is extended by showing that in the asymptotic limit *Ro*, Fr→0, there are independent cascades for the geostrophic and ageostrophic components of the flow, with the latter cascading its energy downscale through catalytic interactions with the former.

11 In this regime, for complex flow patterns, an evolution towards *Ro*,*Fr*∼1 is expected, thus evolving towards an advective breakdown of momentum-balance (§6d).

12 The Stokes–Coriolis force has a relative correction amplitude ∼(*V*_s_/*V*_*g*_)=(*u*_*_/*V*_*g*_) *La*^−2^ with *La*^−2^≫1, which is usually at least as large as *ϵ* in (5.6). By itself it mainly gives rise to an anti-Stokes increment in the Eulerian current velocity, **u**≈−**u**_s_, and otherwise does not greatly disrupt the SMC evolution.

## Data accessibility

This work does not have any data to report.
